# Affinity proteomic dissection of the human nuclear cap-binding complex interactome

**DOI:** 10.1093/nar/gkaa743

**Published:** 2020-09-22

**Authors:** Yuhui Dou, Svetlana Kalmykova, Maria Pashkova, Mehrnoosh Oghbaie, Hua Jiang, Kelly R Molloy, Brian T Chait, Michael P Rout, David Fenyö, Torben Heick Jensen, Ilya Altukhov, John LaCava

**Affiliations:** Department of Molecular Biology and Genetics, Aarhus University, Aarhus, Denmark; Skolkovo Institute of Science and Technology, Moscow, Russia; Moscow Institute of Physics and Technology, Dolgoprudny, Russia; Laboratory of Cellular and Structural Biology, The Rockefeller University, New York, USA; European Research Institute for the Biology of Ageing, University Medical Center Groningen, University of Groningen, Groningen, The Netherlands; Laboratory of Cellular and Structural Biology, The Rockefeller University, New York, USA; Laboratory of Mass Spectrometry and Gaseous Ion Chemistry, The Rockefeller University, New York, USA; Laboratory of Mass Spectrometry and Gaseous Ion Chemistry, The Rockefeller University, New York, USA; Laboratory of Cellular and Structural Biology, The Rockefeller University, New York, USA; Department of Biochemistry and Molecular Pharmacology, Institute for Systems Genetics, NYU Langone Health, New York, USA; Department of Molecular Biology and Genetics, Aarhus University, Aarhus, Denmark; Moscow Institute of Physics and Technology, Dolgoprudny, Russia; Laboratory of Cellular and Structural Biology, The Rockefeller University, New York, USA; European Research Institute for the Biology of Ageing, University Medical Center Groningen, University of Groningen, Groningen, The Netherlands

## Abstract

A 5′,7-methylguanosine cap is a quintessential feature of RNA polymerase II-transcribed RNAs, and a textbook aspect of co-transcriptional RNA processing. The cap is bound by the cap-binding complex (CBC), canonically consisting of nuclear cap-binding proteins 1 and 2 (NCBP1/2). Interest in the CBC has recently renewed due to its participation in RNA-fate decisions via interactions with RNA productive factors as well as with adapters of the degradative RNA exosome. A novel cap-binding protein, NCBP3, was recently proposed to form an alternative CBC together with NCBP1, and to interact with the canonical CBC along with the protein SRRT. The theme of post-transcriptional RNA fate, and how it relates to co-transcriptional ribonucleoprotein assembly, is abundant with complicated, ambiguous, and likely incomplete models. In an effort to clarify the compositions of NCBP1-, 2- and 3-related macromolecular assemblies, we have applied an affinity capture-based interactome screen where the experimental design and data processing have been modified to quantitatively identify interactome differences between targets under a range of experimental conditions. This study generated a comprehensive view of NCBP-protein interactions in the ribonucleoprotein context and demonstrates the potential of our approach to benefit the interpretation of complex biological pathways.

## INTRODUCTION

All RNAs transcribed by RNA polymerase II (RNAPII) are modified at the 5′-end early during transcription with an N7-methylguanosine (m^7^G) linked in a 5′-to-5′ orientation (1,2). The resulting m^7^G-cap structure is bound by the nuclear cap-binding complex (CBC), a heterodimer of NCBP1 (CBP80) and NCBP2 (CBP20) (3). NCBP2 binds directly to the cap, albeit with relatively low affinity; its cap-binding affinity is significantly enhanced by its heterodimerization with NCBP1 (4,5), which further serves as a binding platform for different proteins that influence the progression of RNAs (i.e. ribonucleoproteins; RNPs) towards productive or destructive fates. Through its diverse protein interactions, the CBC is known to modulate various activities of RNAPII transcripts. During transcription, the CBC interacts with P-TEFb and promotes transcription elongation (6); it also interacts with the U4/U6•U5 tri-snRNP to stimulate pre-mRNA splicing (3,7). ARS2 (SRRT, Uniprot gene symbols preferentially used throughout) joins the CBC, forming the CBC-ARS2 (CBCA) complex, which influences the fate of multiple types of RNAs (8,9). CBCA can interact with ZC3H18, which may in turn recruit the nuclear exosome targeting (NEXT) complex or the poly(A) tail exosome targeting (PAXT) connection, directing bound RNAs to decay via the RNA exosome (10,11). On snRNAs and a few independently transcribed snoRNAs, the CBCA complex may interact with PHAX, forming the CBCAP complex which stimulates nuclear export of snRNAs and the movement of snoRNAs to nucleoli (9,12–14). Within elongating (messenger) mRNPs, the CBC interacts with ALYREF in the ‘transcription/export’ (TREX) complex, promoting mRNA export (15).

**Figure 1.**
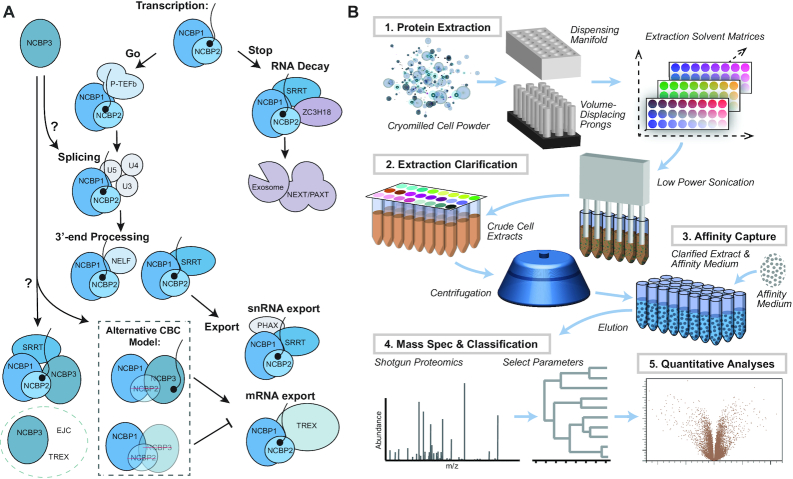
Summary of NCBP interactions and methodological approach. (**A**) NCBP interactions. Right-side pathway: The canonical CBC consisting of NCBP1 and NCBP2 engage in early interactions guided by transcription, splicing, and 3′-end processing, to RNA export or decay (example interactors are shown for each summarized process; see main text for details). Left-side pathway: Putative NCBP3 functional interactions include: participation in splicing (16–18,20); formation of an alternative CBC with NCBP1; and contributions to mRNA export (16). NCBP3 interacts with CBC and ARS2 (5,16) and is affiliated with the EJC, and TREX complexes (16–18,20). (**B**) Methodological approach. Cryomilled cell powders are distributed with a dispensing manifold and macromolecules are extracted with 24 different extraction solutions (1). Brief sonication is applied to disperse and homogenize the extracts (2). After clarifying the extracts by centrifugation, affinity capture is performed (3) and protein eluates are subjected to MS analysis (4) and data processing (5).

NCBP3 (5,16), previously coined C17orf85 or ELG (17–19), was recently proposed to form an alternative CBC with NCBP1, capable of substituting for NCBP2 and suppressing the mRNA export defect caused by loss of NCBP2 (16). Previous reports described the association of NCBP3 with mRNPs to be splicing-linked, exon junction complex (EJC) independent and CBC-dependent (17); yet, NCBP3 has been grouped with the EJC and the TREX complex on the basis of protein-protein interaction studies (18–20). Most recently, NCBP3 was shown to interact *in vitro* with both CBC (via NCBP1) and SRRT, separately and as a ternary complex (5). The complex composed of CBC, SRRT and NCBP3 was shown to be mutually exclusive with PHAX and proposed to be part of an RNA-fate decision tree - similar decision forks between CBC, NELF-E or SRRT (5), and between SRRT, PHAX or ZC3H18 (8) have also been reported. Based on the above mentioned studies, Figure 1A illustrates an abridged narrative for proposed NCBP1, NCBP2 and NCBP3 interactions.

Affinity capture, chiefly immunoprecipitation (IP), is arguably the most popular approach for characterizing target proteins’ physiological interacting partners. However, despite its common use, common implementations of this technique often suffer from shortcomings that include suboptimal parameterization of isolation conditions. For example, the use of salts, detergents, and additives (and mixtures thereof) that:

- do not effectively extract macromolecules affiliated with the target protein, e.g. sending them to the pellet during centrifugal clearance of the extracted solution
- do not stabilize *in vivo* interactions once extracted *in vitro*, causing false negatives, e.g. the apparent *K*_d_ of each co-immunoprecipitated interaction is influenced by the solution character and the potential for synergy with neighboring, cooperative interactions
- or, relatedly, drive the formation of spurious interactions, post-lysis, causing false positives

These kinds of optimizations also apply to other aspects of the IP protocol, including but not limited to the physical treatment during extraction (e.g. mixing/vortexing, sonication etc.), time of protocol and time of incubation with the affinity medium, the physicochemical properties of the tubes and affinity medium, and so on. Hence, significant improvements in performance may be obtained by customizing and optimizing the protocol at critical points (21–26). To tap into the full potential of the technique, we leveraged lessons learned from these examples and developed a platform to parallelize immunoprecipitation while screening performance (27). Thus, this procedure is a mode of quality assurance. A major advance for the technique was the exploration of diverse protein extraction and capture conditions in a manner conceptually similar to crystallographic screening (28), used with comparable rationale and providing comparable benefits to those leveraged by crystallographers; details are illustrated in Figure 1B. In the present study, we retooled the screen for label-free quantitative mass spectrometry (LFQ MS, e.g. reviewed in (29)). To expand our knowledge of the protein-protein interactions exhibited by NCBPs, we carried out a comparative analysis of proteins that co-IP with NCBP1, -2 and -3 from HeLa cell extracts under a variety of experimental conditions.

## MATERIALS AND METHODS

### Preparation HeLa cells expressing LAP-tagged NCBPs and affinity capture

HeLa Kyoto cell lines stably expressing LAP-tagged proteins (NCBP1, 2 and 3) and control, ‘tag-only’ (LAP-control), respectively, were provided as stably transfected cell pools by Ina Poser and Anthony Hyman (see Supplemental Methods, Section 1); these were engineered as previously described (30,31), containing a TEV cleavage site, S peptide, a PreScission protease cleavage site, and EGFP. For C-terminally tagged NCBPs, the LAP-tag DNA sequence is positioned in front of the stop codon; and for the LAP-control cells, the LAP tag DNA sequence is placed under control of the TUBG1 promoter (30). All cell pools provided were FACS sorted for EGFP-positive cells (forward scatter threshold = 5000). The sorted cell pools were cultured using standard techniques in DMEM supplemented with FBS and penicillin-streptomycin. Cell harvesting and cryomilling was carried out as previously described (24). ∼35 mg of cell powder (wet cell weight equivalent) was used per well (NCBP1, 2 and cognate controls), extracted in 450 μl of solution (∼1:13 [w:v]) in 24-wells of a 96-well plate; 300 mg of cell powder was used per affinity capture replicate of NCBP3 and cognate controls at 1:4 (w:v) when conducted individually in microfuge tubes. Screens were conducted as in (27), with modifications described in this study. Sonication was achieved using a QSonica Q700 equipped with an 8-tip microprobe (#4602), applied until material in multi-well plates was homogeneously resuspended as judged by visual inspection (4°C, 1 A, ∼30–40 s [continuous]: ∼140 J per row on average); or using a QSonica S4000 equipped with a low-energy microprobe (#4717), applied individually in microfuge tubes (4°C, 2 A, 15 × 2 s pulses [1 sec interval]: ∼50 J per sample). After centrifugal clarification of the extracts (10 min, 4°C, 20 000 RCF), affinity capture was achieved using 5 μl of affinity medium slurry, conjugated with llama α-GFP polyclonal antibody (25,32), in multi-well screens, or 10 μl of slurry in microfuge tubes. Affinity capture was allowed to proceed for 30 min at 4°C with gentle mixing. Elution from the affinity medium was achieved using 1.1× LDS (ThermoFisher Scientific #NP0008). Protein extraction solutions used and obtained SDS-PAGE, protein staining results are presented in the file: Supplemental_Data_Tables.xlxs; these are also curated in an interactive, searchable form on www.copurification.org (select: ‘search public gels’ → species: human; tagged protein: nuclear cap binding protein subunit [1, 2 or 3]; select ‘next’ → proceed to select additional search options and view output).

### IP-MS experimental design and data processing

**Figure 2.**
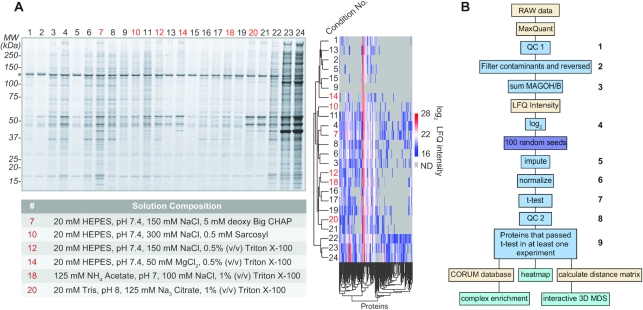
IP-MS pre-screen of NCBP1-LAP and depiction of data processing. (**A**) NCBP1-LAP pre-screen. Sypro Ruby stained SDS-polyacrylamide gel of NCBP1 co-IPs (left) and hierarchical clustering of the cognate MS data using log_2_ LFQ intensity (right). Gray coloring indicates a protein was not detected (ND). Six conditions, highlighted with red lane numbers, were selected for subsequent quantitative screening; detailed solution compositions are listed in the table below the gel. (**B**) Bioinformatic pipeline. After conducting an interactome screen on all three NCBPs and controls - using the conditions highlighted in panel A - the raw MS data were processed in MaxQuant (73) followed by post-processing (see Methods), summarized as follows: (1) inspected PTXQC quality control reports (74); (2) remove common contaminants and reversed protein sequences (FDR-control); (3) merged MS intensities for homologs MAGOH and MAGOHB; protein LFQ intensities were (4) log_2_ transformed, used to (5) impute missing values (multiple imputations with 100 random seeds) and (6) normalized to GFP intensities; (7) results from NCBP and control IPs were compared statistically and (8) imputation-derived false positives were removed; (9) proteins passing a t-test were visualized by using complex enrichment (Figure 3A), heatmaps (Figure 3B), and multidimensional scaling (Figure 5).

The experimental schema is depicted in Figure 1B. Twenty four unique solutions were used for affinity capture pre-screening of NCBP1-LAP. We performed hierarchical clustering of the protein LFQ intensities obtained (Figure 2A); missing values were imputed to 0. To preserve a diverse parameter space for screening while freeing up bandwidth for replicates, we selected 6 extraction and capture solutions from across the breadth of the dendrogram while accounting for the quality of gel profiles (by visual inspection) and proteinaceous complexity (MS-based). These were used in quadruplicate IP-MS experiments for all affinity capture targets (using 24-wells of 96-well plates). Of these, at least three replicates passed initial gel-based quality control (QC) by visual inspection: those lanes exhibiting a relatively discrete pattern of sharply stained bands, a relative paucity of faint/fuzzy background staining, and a band that apparently corresponds to the target protein molecular mass, were moved forward to quantitative MS analyses. Subsequent computational QC of the MS data was applied using the R package PTXQC and in-house R scripts (see *Data processing*, below); NCBP3-LAP multi-well format IP-MS data did not pass QC, and was omitted (see Supplemental Methods, Section 2); poor quality was attributed primarily to low yield for this target protein at the 35 mg-scale used for multi-well IPs. To obtain usable IP-MS data for this target, the scale was increased to 300 mg and repeated in microfuge tubes, in four extraction and capture solutions and three replicates each. For multi-well and microfuge tube experiments, NCBP and LAP-control targets were captured under identical conditions, respectively. Accounting of all the samples and replicates used in this study is provided in the file: Supplemental_Data_Tables.xlxs. Statistical methods are described in detail, below.

### Sample workup and mass spectrometry

Samples were reduced (DTT) and alkylated (iodoacetamide), and a fraction of the sample was subjected to standard SDS-PAGE, staining with Sypro Ruby, and CCD imaging (Fuji LAS-4000); the other fraction was run as a gel plug, Coomassie Blue stained, excised, subjected to in-gel tryptic digestion, and the peptides desalted and concentrated upon C18 resin (OMIX C18 pipette tips; Agilent #A57003100) essentially as previously described (33). For multi-well screens, $\frac{1}{2}$ of the sample was used for gel-based analysis and MS, respectively. For NCBP3 IPs done in microfuge tubes, ⅙ of the sample was used for gel-based analysis and the rest for MS. Standard SDS-PAGE was used for initial QC of sample composition and excision of select bands (based on imaging) for protein identification by tandem mass spectrometry; gel plugs were used to prepare whole IP fractions for LFQ MS. Summarized as follows: Samples produced by multi-well screening were run on either an Orbitrap Fusion or a Q Exactive Plus (Thermo Fisher Scientific). Dried peptide samples were resuspended in 10 μl of 5% (v/v) methanol, 0.2% (v/v) formic acid in water; half was loaded on the LC column (Thermo Easy-Spray ES800). Peptides were ionized by electrospray at 1.8–2.1 kV following elution across a linear gradient (7 min for individual gel bands; 35–40 min for whole gel plugs) rising to 30% (v/v) acetonitrile. Solvent A was 0.1% (v/v) formic acid in water; Solvent B was 0.1% (v/v) formic acid prepared by combination with either 95% (v/v) or neat acetonitrile. Full MS scans were performed in profile mode, while fragmentation spectra were acquired in centroid mode with priority given to the most intense precursors. Dynamic exclusion was enabled to limit repeated sequencing of the same peptides. For NCBP3-LAP and control samples obtained by microfuge tube affinity capture: the dried peptide mix was reconstituted in a solution of 20 ul of 2% (v/v) formic acid (FA) for MS analysis. 5 μl of this solution was loaded with the autosampler directly onto a self-packed column, which was made from a 75 μm ID PicoFrit column (New Objective, Woburn, MA, USA) filled with 25 cm of 2.4 μm Reprosil-Pur C18 AQ. Peptides were eluted at 200 nl/min from the column using an Eksigent NanoLC 415 with a 52 min gradient from 2% to 25% buffer B (0.1% (v/v) formic acid in acetonitrile); at which point the gradient was switched from 25% to 85% buffer B over 5 min and held constant for 3 min; finally, the gradient was changed from 85% buffer B to 98% buffer A (0.1% (v/v) formic acid in water) over 1 min, and then held constant at 98% buffer A for 15 more minutes. The application of a 3.5 kV distal voltage electrosprayed the eluting peptides directly into a Q Exactive HF mass spectrometer equipped with an EASY-Spray source (Thermo Scientific). Mass spectrometer-scanning functions and HPLC gradients were controlled by the Xcalibur data system (Thermo Scientific). The mass spectrometer was set to scan MS^1^ at 60,000 resolution with an AGC target set at 3 × 10^6^. The scan range was *m*/*z* 375–2000. For MS^2^, resolution was set at 15,000 and AGC target at 2 × 10^5^ with a maximum IT at 50 ms. The top 15 peaks were analyzed by MS^2^. Peptides were isolated with an isolation window of *m*/*z* 1.6 and fragmented at 27 CE. Minimum AGC target was at 8 × 10^3^. Only ions with a charge state of 2 through 6 were considered for MS^2^. Dynamic exclusion was set at 15 sec. See Supplemental Methods, Section 3 for an ordered summary of instrument settings.

### Data processing

**Figure 3.**
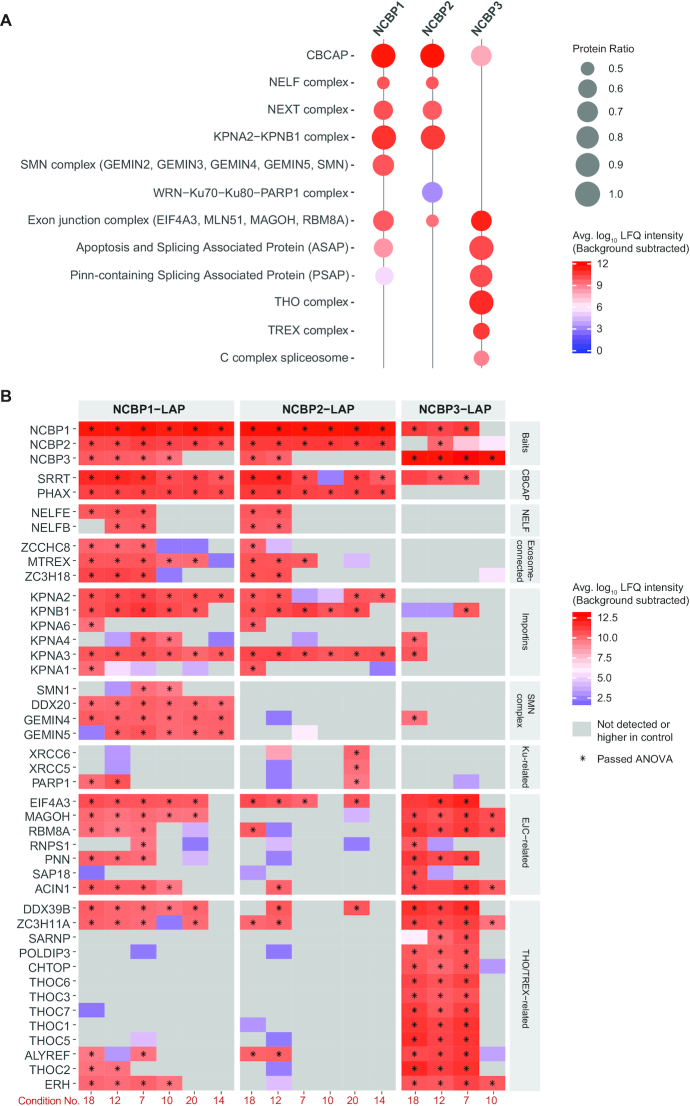
Complex- and protein-centric data visualizations. (**A**) Protein complex enrichment plot. A composite representation of protein complexes observed across all IP-MS experiments, combined for each NCBP. Complexes were only represented in this plot if half or more of their constituent components passed statistical cut-offs (adjusted *P*-value ≤ 0.05 and log_2_ fold change ≥ 1) within the screen. This is represented by the ‘protein ratio.’ A minimum protein ratio of ≥ 0.5 was enforced for complexes with three or more components; for two-component complexes, both components must be present. Complex annotations were taken directly or modified from CORUM to avoid redundancy (36). The relative abundance of each complex captured is given as the control subtracted, average LFQ intensity of the constituent proteins (see Methods). Complex LFQ intensities are presented as their log_10_ values. A detailed interactive data interface, including results isolated from specific IP-MS experimental conditions and all Uniprot IDs for complex membership versus those identified, is available at https://ncbps.shinyapps.io/complex_enrichment/; comparable information is also provided in the file: Supplemental_Data_Tables.xlxs. (**B**) Individual protein enrichments. A summary heatmap with a focus on individual proteins, grouped based on selected CORUM complexes (manually inspected) and annotated protein functions. Control subtracted, average LFQ intensity of the quantified proteins are presented as their log_10_ values; negative or zero intensities are in grey. Proteins that passed the significance threshold (adjusted *P*-value ≤ 0.05 and log_2_ fold change ≥ 1) are marked with asterisks.

Our computational proteomic pipeline is summarized in Figure 2B. Pre-processing of raw data was done in MaxQuant; data post-processing, statistical filtering, and distance calculations were done in R—all code is available at https://github.com/moghbaie/NCBP-pipeline. Peptide identification and quantitation was achieved using the MaxQuant v.1.6.5.0 software and a proteomic database comprised of a Uniprot human proteome (proteome:up000005640; reviewed:yes) with GFP added (97% identical to the EGFP sequence in the LAP-tag). The following abridged software settings were used: include contaminants—true; PSM & protein FDR—0.01; quantify unmodified peptides and oxidation (M), acetyl (protein N-term), carbamidomethyl (C); phospho (STY) was searched but not quantified, nor were unmodified counterpart peptides; iBAQ—true; iBAQ log fit—false; match between runs—true; decoy mode—revert; Include contaminants—true; advanced ratios—false; second peptides—true; stabilize large LFQ ratios—true; separate LFQ in parameter groups—true; require MS/MS for LFQ comparisons—true; razor protein FDR—true. The RAW and MaxQuant v.1.6.5.0 processed files are available for download via ProteomeXchange with identifier PXD016038. Data quality was initially assessed using the output from MaxQuant with the R package PTXQC (33) and in-house R scripts - examples are detailed in the Supplemental Methods, Section 2. Proteins marked as ‘contaminants’ or ‘reverse’ by MaxQuant were removed and intensities of the proteins MAGOH (UniProt ID P61326) and MAGOHB (UniProt ID Q96A72) were summed together in every experiment (see Supplemental Methods, Section 4). Only proteins which had ‘Peptide counts (razor + unique)’ ≥2 were retained for analysis. Using the data obtained, protein intensities were log_2_-transformed in order to sample from a normal distribution during imputation (see Supplemental Methods, Section 5 for a full description with performance testing and results). Imputation for LFQ and iBAQ intensities was repeated 100 times (see below). To compare protein LFQ or iBAQ intensities between different experiments, they were normalized by the values for GFP obtained from within the same experiment (see Supplemental Methods, Section 6). For statistical testing, the intensities were rescaled by multiplying all values by a coefficient equal to the mean of the smallest and the largest intensity value before the normalization, restoring the original range of the data; log_2_-transformed intensities were used for further analysis. Proteins were subjected to unpaired, two-sample t-tests between LAP-tagged-targets and LAP-only-controls for each set of IPs. Protein enrichment with the LAP-tagged NCBP target in each experiment was considered statistically significant if the Benjamini–Hochberg adjusted *P*-value was ≤0.05 and log_2_ transformed fold-change (log_2_FC; target/control) was ≥1. To mitigate imputation-induced sampling artifacts, only proteins that reached significance in ≥ 60 (of 100) imputation trials were retained for further consideration (see Supplement Methods, Section 6). Precise adjusted *P*-value and fold-change metrics can be found in the file: Supplemental_Data_pvalues_logfc.xlsx. Next, all proteins reaching significance in at least one experiment were collated: these values were used in quantitative analyses. For every protein reaching statistical significance for co-enrichment with an NCBP target at least once, pairwise euclidean distances were calculated. For Figure 3 (complex enrichment plot and heatmap), background subtracted LFQ intensities were used to eliminate contributions by non-specific interactions. This was achieved by subtracting the mean LFQ intensities of proteins in control experiments from the LFQ intensities in the NCBP IPs, after which, the values were log_10_-transformed; the subtraction was applied to each experimental condition separately.

## RESULTS

### Interaction screen and data analysis scheme

In our previous work, we primarily distinguished differences in the compositions of affinity captured protein complexes through the visual examination of Coomassie Brilliant Blue-stained SDS-polyacrylamide gels, followed by identification of the differentially enriched protein components by MALDI-TOF MS. This approach was successful on several multi-protein complexes we chose to examine (27), including those that form RNPs and/or metabolize RNA, and has enhanced our discovery potential on additional projects since (e.g. (34,35)). The prior implementation, however, proved most suitable for the study of affinity enriched protein complexes that exhibited high enough yield of their individual constituent proteins to detect differences by general staining, visually. We did not readily observe these characteristics when applying a 24-condition screen to NCBPs; exhibited for NCBP1 in Figure 2A. Thus to conduct a thorough study of NCBP interactomes, we modified and updated our procedure to: (i) pre-screen for experimental conditions that maximize sample diversity across a given space, (ii) accommodate control experiments and sufficient replicates for LFQ MS analysis of immunoprecipitated fractions, and (iii) provide bioinformatic resources useful for parsing and exploring interactome differences, statistically.

After conducting an initial 24-condition IP/MS ‘pre-screen’ of NCBP1-LAP (localization and affinity purification tag; using anti-GFP affinity medium, see Methods), six conditions were chosen as exemplars for quantitative analysis. Figure 2A shows how the proteomic profiles obtained after IP compared in terms of proteomic-complexity and abundance as visualized by SDS-PAGE and Sypro Ruby staining (left panel) or the hierarchical clustering of LFQ intensities obtained upon analysis of resulting MS data in MaxQuant (right panel; see Materials and Methods). This allowed us to semi-quantitatively pick conditions spread across the dendrogram, aided by qualitative visual inspection of the gel, for the purposes of optimizing sample proteomic diversity in subsequent quantitative analyses, while freeing up bandwidth for IP replicates required for statistical sample comparisons. Note: the extremes of the dendrogram were avoided due to (i) exhibiting poor relative enrichment of NCBPs compared to many other proteins (conditions 22–24, have telltale signs of high non-specific background) and (ii) exhibiting few other proteins that were not also encompassed by other profiles (e.g. conditions 1, 2, 5, 13).

Thereafter 6-condition screens, with four replicates per condition, were applied to all LAP-tagged NCBPs and cognate controls (LAP-tag only) in anti-GFP IPs. Analysis of NCBP3-LAP demonstrated that the yield of this protein did not reach comparable levels to NCBP1- and 2-LAP at the standard scale used in the screen; therefore, to more accurately assess the NCBP3-LAP interactome, we increased the scale and repeated IP-MS across four conditions, in three replicates each (see Methods). Once a complete experimental dataset for all three NCBPs was obtained, we applied the bioinformatic analyses outlined in Figure 2B. To enhance performance, we developed a novel approach to impute missing values in our data, a common challenge in LFQ MS (see Methods and Discussion). After statistical analysis was performed to determine which proteins were enriched in the NCBP-LAP IPs compared to controls, further analyses leveraging various visualization techniques were explored in an effort to reveal and emphasize differences in NCBP interactomes, presented below.

### A complex-centric view of NCBP differences

To obtain a general, first-pass comparison of our samples, we explored the overlap between our NCBP data and protein complexes curated in the CORUM database (36). Figure 3A (‘complex enrichment plot’) displays a composite summary of the differences in protein complexes affinity captured with LAP-tagged NCBPs, including those illustrated in Figure 1A. The plot sacrifices details concerning the individual components of each complex in order to provide a functional summary of macromolecular differences.

In the literature, NCBP1 and 2 are largely described in the context of their cooperative activities as the CBC; represented here by interactions with e.g. CBCAP in sn(o)RNA transport (9), NELF (negative elongation factor) in 3′ end processing (37), NEXT in exosomal RNA decay (10), and the karyopherin import complex KPNA2-KPNB1 in the cytoplasmic-nuclear recycling of the CBC (38). However, we also observed potential independent/preferential protein complex associations for each: survival of motor neurons (SMN) complex, involved in snRNP biosynthesis and assembly (39), was co-enriched only with NCBP1. Some DNA-related complexes preferentially co-enriched with NCBP2, compared with NCBP1, such as the WRN−Ku70−Ku80−PARP1 complex (40). Moving to NCBP3, we observed that it was neither appreciably associated with NELF (previously reported (16)) nor RNA exosome-related complexes (i.e. NEXT). Instead NCBP3 was more appreciably associated with EJC and EJC-affiliated complexes (ASAP, PSAP) and was even more contrastingly associated with THO (suppressors of the transcription defects of hpr1Δ mutants by over-expression) and TREX complexes as well as the spliceosomal C complex. The differential associations of NCBP1, 2, and 3 with EJC and THO/TREX are cross-validated and functionally dissected in a separate manuscript (41). Additional complexes are presented in a detailed interactive collection of enrichment plots online: https://ncbps.shinyapps.io/complex_enrichment/. In the online interface, results can be sorted by target (e.g. as presented in Figure 3A) or by capture condition to display precisely which conditions support the co-IP of which associated complexes for each NCBP target.

To reveal finer protein-level details, the heatmap shown in Figure 3B was generated: NCBP1-LAP co-captures NCBP2 and vice versa in all conditions tested, and NCBP1-LAP co-captures NCBP3 in four out of six conditions (adjusted *P*-value ≤ 0.05 and log_2_ transformed fold-change ≥ 1, see Materials and Methods; precise adjusted *P*-value and fold-change metrics can be found in the file: Supplemental_Data_pvalues_logfc.xlsx). NCBP2-LAP co-captures NCBP3 in conditions 18 and 12, and NCBP3-LAP co-captures NCBP2 in condition 12 but co-captures NCBP1 in conditions 18, 12, and 7; although NCBP3 also co-captured NCBP2 in conditions 7 and 10, these interactions did not surpass statistical cut-offs for co-enrichment in those conditions. Because NCBP2-LAP co-IPs NCBP3 and NCBP3-LAP reciprocally co-IPs NCBP2, in both cases along with NCBP1, these data support a model where NCBP3 association with NCBP1 (or, more precisely, NCBP1 affiliated macromolecules) is not mutually exclusive with NCBP2. NCBP1 and NCBP2 are always strongly co-associated, but their associations with NCBP3 appear to be less stable and the connection is lost in several conditions. All three NCBPs were associated with the protein SRRT, but only NCBP1 and NCBP2 were also observed to be associated with PHAX (confirming previous reports (5,16)). An extended supplemental heatmap (Supplemental_heatmap.pdf) displays additional proteins, including spliceosomal proteins.

### Re-examining the relative abundance of NCBP1:NCBP2

NCBP1 and NCBP2 were robustly co-associated across all NCBP1/2 IPs, yet their co-IP profiles also exhibited features distinguishing them from one another. Perhaps, outside of the context of the CBC, NCBP1 and/or NCBP2 participate in the formation of independent macromolecules. We reasoned that support, or opposition, for this idea may arise by examining the relative abundance of NCBPs present in their reciprocal IPs.

**Figure 4.**
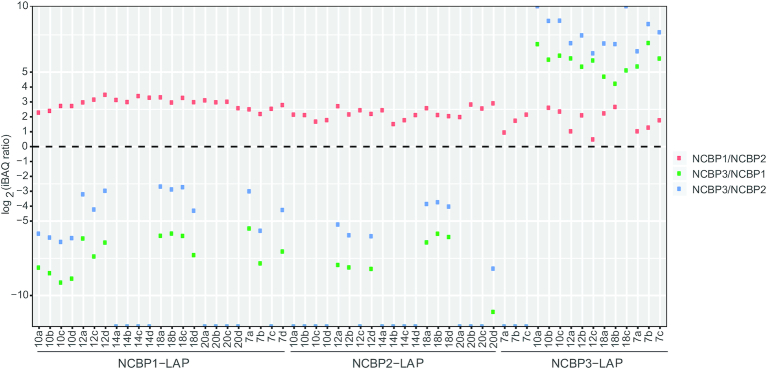
Ratios of NCBPs in individual IPs. The log_2_ ratios of the NCBP iBAQ intensities were calculated for each IP experiment as a proxy for relative copy number—shown as NCBP1/NCBP2 (red), NCBP3/NCBP1 (green), NCBP3/NCBP2 (blue). NCBP1 has the highest copy number in NCBP1-LAP IPs and NCBP2-LAP IPs, while NCBP3 has the highest copy number in NCBP3-LAP IPs.

To address this question, we plotted NCBP MS-derived iBAQ intensity ratios, as displayed in Figure 4. iBAQ intensity ratios can be used as a proxy for protein proportions because they are normalized by the number of theoretically observable peptides for each protein (42); larger proteins encompass a greater number of observable peptides, smaller proteins, fewer. Examining the relative abundances of NCBPs revealed that the relationship between NCBP1 and NCBP2 was atypical. NCBP3 IPs illustrate why this is so: NCBP3 is more abundant than NCBP1 and NCBP2 when NCBP3 is the IP target (Figure 4, right side of plot). The target protein nearly always exhibits apparent superstoichiometry to its *in vivo* interaction partners in IP fractions; among other possibilities, in a well optimized IP this can usually be attributed to the antibody-antigen interaction being the highest affinity interaction in the mixture. This is also the reason why the same protein (initial target) then becomes substoichiometric in reciprocal IPs, that are commonly used to confirm IP-based interactions. In line with this expectation, and as mentioned earlier, NCBP3 is less abundant, or missing, in IPs targeting NCBP1 and NCBP2.

When NCBP1 is the IP target, it also exhibits apparent superstoichiometry. However, NCBP2 exhibited a surprising trend as the target of IP: NCBP2 appears substoichiometric to NCBP1, exhibiting several-fold lower abundance. This trend is prevalent across all the NCBP2 IPs and suggests that while NCBP1/2 stably interact (the expected result), NCBP1 might be present in more than one copy per NCBP2 in an RNP context (see Discussion).

### Contextualization of NCBPs in mRNP maturation

**Figure 5.**
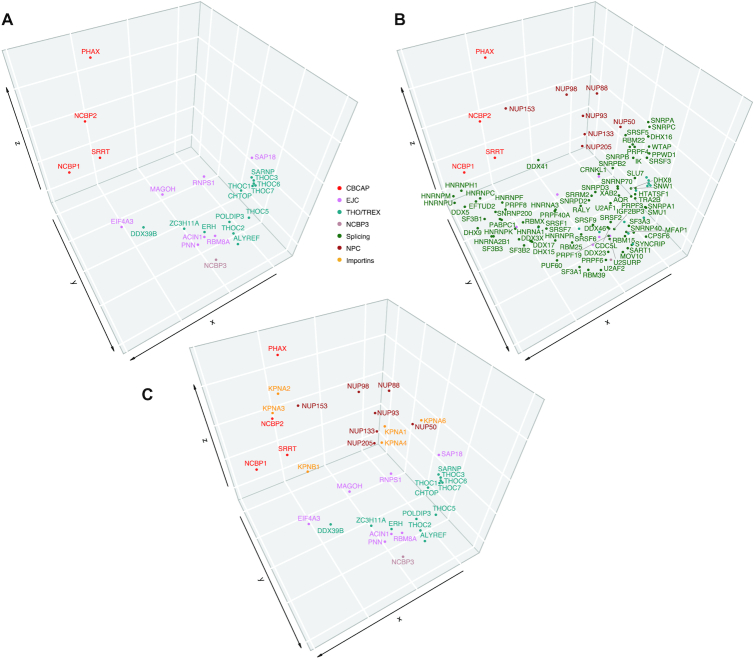
Global analysis contextualizes NCBP interactor relationships - multidimensional scaling (MDS). Select protein complexes are shown in a static 3D MDS plot (interactive plot: https://ncbps.shinyapps.io/3d_mds_app/). This is a composite analysis of all IPs and all interacting proteins. Three panels selectively display some groups while omitting others for clarity in reading the figure labels but all nodes are simultaneously present in the analysis at the positions indicated. The colour scheme for different groups of proteins is illustrated in the center of the plot. Panel (**A**) displays the segregation of the NCBPs, SRRT, PHAX, the EJC, and THO/TREX proteins. Panel (**B**) labels, CBCAP, spliceosomal, and NPC proteins with text; the nodes displayed for NCBP3, EJC, and THO/TREX in panel (A) are also included in (B) for positional context, without labels to avoid crowding. These complexes can also be viewed in 3D context with rotation in the supplemental file: 3D_Animated_MDS.gif. Panel (**C**) displays the segregation of the NCBPs, SRRT, PHAX, the EJC, THO/TREX, NPC, and importins.

We sought to integrate and parse all the measured protein behaviors to gain a global view of protein interrelationships across IP targets and capture conditions. To achieve this we performed a multidimensional scaling (MDS) distance analysis and created an interactive 3D MDS plot (https://ncbps.shinyapps.io/3d_mds_app/; see Methods). Three representative snapshots of the plot are displayed in Figure 5. This analysis visually conveys the measured associations of the proteins across the co-IP-MS experimental space: each experimental replicate contributes a dimension and MDS places each protein in a lower dimensional space that conserves distances between proteins as much as possible. In the present case, we had 57 total dimensions before reduction, composed of 23 NCBP1-LAP IPs, 22 NCBP2-LAP IPs and 12 NCBP3-LAP IPs. The output permitted us to compare and contrast the segregation of proteins that exhibited one or more statistically significant enrichments by co-IP (P-value ≤ 0.05, log2 fold-change ≥ 1) within the parameter space explored in the screen. The settings on the interactive plot allow users to hide or show different groups of proteins and also to calculate the MDS distance of proteins that passed from 1 to 12 statistical significance tests for co-enrichment from the full set of NCBP IPs.

Considering all IPs conducted in this study, only NCBP1/2 (CBC), SRRT, PHAX, KPNA3 and HNRNPUL2 passed our threshold for specific enrichment twelve separate times. CBC, SRRT and PHAX are established interactors as part of the CBCA and CBCAP complexes (8–10). KPNA3 (importin subunit alpha-4) has been suggested to interact with both NCBP2 and NCBP3 (16). In our MDS analysis (Figure 5C), KPNA2, KPNA3 (importin-α) and KPNB1 (importin-β) cluster proximal to NCBP2 and NCBP1 (whereas NCBP3 clusters apart). The CBC-importin-α complex binds capped RNA in the nucleus, and the binding of importin-β stimulates the release of capped RNA from the CBC-importin-α complex in the cytoplasm (38,43). We identified KPNA3, KPNA2, KPNB1, and RAN among these IP (see Figure 3B and Supplemental_heatmap.pdf), likely representing the different forms of CBC/RNP-importin associations. HNRNPUL2 has not previously been reported as an interactor of NCBPs, and very little information is available about this protein, but given these data, a functional role in coordination with CBC is likely.

Using the default settings (all groups, each protein must exhibit statistical significance for enrichment at least once) some observations appeared striking: (i) the CBC, SRRT and PHAX segregate to a region of the graph that is otherwise relatively sparse of other nodes at a common extremity of the MDS plot. NCBP1/NCBP2/SRRT triangulate one another, with PHAX located on the opposite side of NCBP2 as NCBP1 and notably distal from the center of mass of the graph (Figure 5A); (ii) splicing proteins form an arch extending from the CBC towards the opposite extremity of the space, terminating near a cluster of nuclear pore complex (NPC) proteins (Figure 5B) and (iii) within this ‘splicing arch,’ located between the CBC and the NPC, EJC proteins are interspersed with THO/TREX components; NCBP3 segregates nearly centrally within this EJC/THO/TREX spread (Figures 5A and B).

Considered directionally, these observations are remarkably consistent with the spatio-temporal features of the RNAPII-transcribed, mRNP maturation pathway. The binding of CBC-connected proteins represents one of the earliest steps in RNP assembly on 5′-capped, RNAPII transcripts (Figure 5A). The CBC proteins present at an interface with splicing proteins, a subsequent step in mRNP maturation (that typically occurs multiple times) - those most proximal to NCBP1 include HNRNPH1, HNRNPM, HNRNPU, HNRNPC, DDX5, and EFTUD2, followed by a myriad of other splicing proteins, spreading across a large, arching space (Figure 5B). This spread of proteins encompasses a collection of biochemically diverse and temporally staggered interactions, of which splicing is known to consist. The proteins we observed constitute an incomplete, cross-sectional composite of up to 79 out of 143 splicing factors curated by merging the CORUM categories ‘spliceosome C complex’ and ‘spliceosome’ - over 170 proteins have otherwise been classified as human spliceosome components (44–49). Towards the middle and end of the region demarcated by splicing proteins, EJC and TREX proteins appear next (text labels in Figure 5A, overlap with splicing proteins in Figure 5B); these are the proteins deposited at exon-exon junctions after splicing and those that shepherd mRNPs from transcription to export. First EIF4A3 and DDX39B appear; then MAGOH, which partitions adjacent to the splicing arch, roughly parallel to ZC3H11A, which lies within the arch; followed by ACIN1, PNN, ERH, NCBP3 and RBM8A partitioned in relative proximity within the crook of the arch, NCBP3 approximately at its apex. These are followed, among other EJC/TREX-linked proteins, by multiple THO complex components (also contributing to mRNP maturation and export; text labels in Figure 5A, overlap with splicing proteins in Figure 5B), reaching a terminal cluster along with various splicing factors. At this end of the arch, and opposite from the CBC, lies a cluster of NPC proteins (NUP50, NUP88, NUP93, NUP98, NUP133, NUP205; Figure 5B), likely representing interactions exporting mRNPs from the nucleus. An exception to this is NUP153, which segregates closer to the opposite side of the arch (Figure 5B): we speculate that this dichotomy reflects (i) import of the CBC through the NPC, via docking to the nuclear basket protein Nup153 (50) (karyopherins / importins also cluster nearby, described above) and (ii) the final stages of nuclear export through the NPC via Nup98 (51,52) and the cytoplasmic export platform that includes Nup88 (53).

## DISCUSSION

### Interactome mapping by IP-MS

Among the most difficult challenges to interactome mapping by co-IP is the parameterization of optimal working conditions, enabling the transfer of target macromolecular complexes out of living cells and into the test tube. This is made more difficult by the fact that, while the target protein is known in advance, the macromolecular assemblies it forms in the cell are rarely known; and are highly unlikely to be known comprehensively, given the present state of interactome incompleteness (54,55). When attempting to IP macromolecular complexes, the sample workup must afford at least the following two features to ensure the results will be informative: (i) the maintenance of at least some physiologically relevant macromolecular configurations and (ii) the simultaneous mitigation of accumulating post-lysis artifacts. Parameter optimization by parallelized IP screening confers access to these two features, representing a practice that provides quality assurance to IP-MS studies (27). In the present study, we chose to apply this approach to chart NCBP interactomes (Figure 1A); these proteins join complex, dynamical RNPs in the cell, presenting a challenging use case. Upon initial screening, we found that the main differences between NCBP IPs were not sufficiently interpretable through the lens of SDS-PAGE/general protein staining alone. Although MS is most often used as an endpoint readout to verify sample composition, in this study we show that when SDS-PAGE protein banding patterns are not highly informative, MS, in conjunction with PAGE, is warranted for quality assurance and parameter selection (Figure 2A). Quantitative analysis using MS, along with appropriate controls, across the selected parameters can then be carried out with confidence (Figures 1B, 3, 4 and 5).

We chose to apply a popular LFQ MS based analysis using MaxQuant and custom post-processing in R (Figure 2B). We chose to use LFQ in-part because it is applicable to patient tissues, where e.g. genomic tagging and metabolic labeling are not possible; it is our intention to apply a comparable approach to clinical samples in future studies. LFQ is also inexpensive but does come with some well-known shortcomings; one of which is the failure to detect common peptides (and thus proteins) between all samples analyzed. Although missing values are an intrinsic property of data dependent LFQ MS, it is notably problematic for the proteins that are enriched during the IP and not present in the cognate control. In a well-optimized IP, many (or most) of the proteins that co-IP with the experimental sample may not be detectably present in the control (24); but because proteins with missing values in the control cannot be expressed in terms of the commonly utilized metric ‘log_2_ fold-change’, imputation of small values to replace the missing values is a frequently used solution (e.g. reviewed in (56–58) and implemented in popular proteome analysis software). However, performance among imputation approaches varies. To improve the performance in our application, we developed a novel approach which takes advantage of information provided by the sample replicates (see Supplemental Methods, Section 5). Our algorithm resembles a decision tree that applies imputation in different ways depending on the degree to which it is needed. It outperformed the default imputation approach used in the popular Perseus software on the data produced in this study. Because imputation may suffer from instance-specific deficiencies, further trial and error testing is needed to determine the broader utility of our algorithm; based on its performance in this study we believe it holds great promise (we also applied it successfully in another LFQ MS study (59)).

### NCBP interactome differences

With a well-performing bioinformatics pipeline in hand, we sought to mine the NCBP interactomes for missing information, potentially adding to our understanding of the macromolecules they form together and/or apart. We achieved this using three main visualizations: complex enrichment plots (Figure 3A), heatmaps (Figure 3B), and MDS (Figure 5). These analyses revealed highly similar protein associations exhibited by both NCBP1 and NCBP2, but also support possible moonlighting (60). NCBP1 IPs highly enriched components from the SMN complex, especially the SMN−independent intermediate containing GEMIN5, GEMIN4, and DDX20 (GEMIN3), but such enrichment was not observed in NCBP2 IPs, suggesting a stronger linkage of NCBP1 than NCBP2 to the snRNP maturation process (Figure 3). We also noticed some DNA-binding complexes preferentially co-enriched with NCBP2 compared with NCBP1 (albeit only captured in one IP condition); these included the Ku-containing and PSF−p54-containing complexes (Figure 3 and https://ncbps.shinyapps.io/complex_enrichment/). This result may implicate NCBP2 in chromatin-associations that are distinct from NCBP1. There is scientific precedent for independent functional roles of NCBP1 and NCBP2: it has been shown that NCBP1 is expressed with roughly three-fold higher abundance than NCBP2 (16), supporting the hypothesis of some independent functions (60). Adding to that: individual depletion of NCBP1 and NCBP2 results in different effects on RNA export (16); in yeast, genetic deletion mutants of Cbp80p (NCBP1 homolog) and Cbp20p (NCBP2 homolog), respectively, share fewer than half of the resulting gene expression-level changes in common (61); and NCBP1, but not NCBP2, was co-enriched with eIF4E-bound transcripts (62), establishing NCBP2-independent, NCBP1-associated RNPs.

NCBP3 has been proposed capable of substituting for NCBP2 in the CBC and suppressing mRNA export defects caused by NCBP2 loss (16). Our findings do not contradict that proposal, but they show that NCBP1, NCBP2, and NCBP3 may normally all be present simultaneously within the same population of isolated RNPs - NCBP2 can co-IP NCBP3 and NCBP3 can co-IP NCBP2 (Figure 3B). If the existing cap-binding paradigm holds, then within mRNPs, NCBP3 may sometimes bind NCBP1/2 (canonical CBC)—fostering crosstalk with other mRNP maturation factors (discussed below). NCBP1 and NCBP2 are thought to be present in a 1:1 stoichiometry in the canonical CBC; yet we observed that NCBP1 may be present in apparent excess to NCBP2 (Figure 4) - importantly, this was also observed in IPs targeting NCBP2. This raises the possibility of multiple copies of NCBP1 in some NCBP1/2 containing macromolecules. Our current findings are not conclusive and warrant deeper follow-up analysis, including more precise and accurate quantitation by alternative methods. But if this observation holds true, our co-IPs of NCBP3 with NCBP2 and NCBP2 with NCBP3 may signify a mixture of interactions: NCBP1 with NCBP2, together in the context of the CBC, and NCBP1 with NCBP3 together in a distinct context, possibly more distal from the 5′-cap—the latter scenario would naturally increase the NCBP1:NCBP2 ratio in some mRNPs. We consider this possibility to be bolstered by knowledge of NCBP1s excess expression to NCBP2 and potential to engage RNPs apart from NCBP2 (16,62).

Before NCBP3 was proposed to bind 5′-caps, its associations with mRNPs were instead linked to splicing and grouped with the EJC and TREX (17–20). Our studies reinforce these associations (Figure 3), placing NCBP3 at an interface between splicing, EJC, and TREX based on MDS analysis (Figure 5). All the evidence considered together, a parsimonious conjecture is that when NCBP3 is present, it (primarily) associates with EJC/TREX and may also (secondarily) interact with CBC via NCBP1 (when PHAX is not present). Notably, NCBP1 exhibits a somewhat intermediate level of EJC enrichment compared to NCBP2 (low EJC) and NCBP3 (high EJC), while NCBP3 associations are also strongly skewed towards THO/TREX.

### MDS reconstructs ordered pathways from heterogeneous interactions

The MDS analysis provided key evidence to encourage our speculations regarding possible CBC and NCBP3 macromolecular organization. Our implementation is agnostic to how many times a protein was statistically significant, as long as it was significant at least once (statistical stringency is user selectable in the interactive MDS, https://ncbps.shinyapps.io/3d_mds_app/). This decision was made using the following rationale: in this study, we first formed our belief in a collection of *in vivo* interactions based on evidence, obtained from across the breadth of our screen, in the form of *t*-tests comparing cases and controls after IP-MS analysis. Thus having a large set of physiologically believable interactions, we then focussed on their *in vitro* behavioral patterns holistically. In a recent study, we showed that the co-partitioning of proteins across numerous IP-MS experiments revealed known and novel physical and function relationships, providing a basis for algorithmic clustering of putative macromolecules from heterogeneous mixtures (63). Although the affinity proteomics and algorithm design used here were distinctive in several details compared to the referenced prior study, comparable concepts apply. In short, it is informative for us to observe the co-behavior of all proteins that interact specifically, even when, in a given experiment they are not shown to be specific in that test tube.

We contend that the clustering of protein behaviors in our MDS plot provides the basis for a ‘pseudo-timeline’ of protein interactions along enriched pathways. MDS and other dimensionality-reduction techniques are already being used for conceptually comparable purposes e.g. in reconstructing the temporal progressions of cell states from transcriptional signatures (64–66). As described in the results section, the MDS graph (Figure 5) revealed a conspicuous and reassuring assortment of proteins that cluster together with other functionally-like proteins; the arrangement of the protein clusters related to mRNP maturation also occur in a manner that recapitulates the accepted composition and order of assembly of the mRNP pathway (67,68). Several striking observations follow: (i) The CBC, SRRT, and PHAX segregate to a region of the graph that is otherwise sparse of other nodes at a common extremity of the MDS plot, with PHAX notably distal from the center of mass of the graph - this distance may be rationalized in light of our panel of results being enriched for mRNP processing factors - whereas PHAX connects with sn/snoRNP processing (12,13); conducting PHAX IPs should contribute a new segment to the plot in this sparsely occupied space; (ii) Splicing proteins form an arch extending from the CBC towards the opposite extremity of the space, terminating in a cluster of NPC proteins; this is satisfyingly consistent with the reconstruction of a pseudo-timeline spanning cap-binding through processing and nuclear export of mRNAs; (iii) Within the ‘splicing arch’, located between the CBC and NPC, EJC proteins interspersed with THO/TREX components appear; NCBP3 segregates nearly centrally within this EJC/THO/TREX spread; supporting the prior art and our contention that NCBP3 is, first and foremost, a physical and functional component of these complexes, rather than the CBC. If NCBP3 joins mRNPs after the canonical CBC has already formed, then the *in vivo* relevance of NCBP3’s ability to bind m^7^GTP *in vitro* (5) remains open for clarification (this binding will have already been satisfied by the CBC). One relevant scenario may be when NCBP3 substitutes for NCBP2, forming an alternative CBC: this may occur only (or primarily) upon NCBP2 loss or down regulation. If so, this activity may be mechanistically distinct from the splicing and mRNP maturation-connected NCBP3 narrative described by others (17–20), and reinforced based on the interactions observed here.

To avoid over-interpreting these data at an unjustified granularity, we are treating the result as largely descriptive. What is clear is that the interrelationships we observed broadly follow the interaction sequence and functional rationale believed to apply *in vivo*; it is notable that comparable though increasingly less complete assortment patterns of protein classes are discernible in the MDS plot when restricting the proteins analyzed only to those that have passed up to 4 t-tests (above which the number of nodes are few and the pattern begins to degrade). That said, these data emanate from a screen that explores macromolecule stability via their differing compositions, obtained under a multitude of biochemical challenges - the relative abundances of the proteins offers an opportunity for quantitative readout of their affinities, given experimental conditions. Interactomics as a discipline has demonstrated the authentic recapitulation of macromolecular compositions by sampling a large number of target proteins using IP-MS (e.g. (69,70)) - but the community also understands that there is still a great deal of missing information (54,55). We contend that this information is missing, in large part, because the conditions of IP-MS are not optimized for the target macromolecules; as a result, *in vivo* interactions are rapidly shed during extraction and thus elude detection (27) - the present study extends the tools at our disposal to combat missing information.

## DATA AVAILABILITY

R code available at https://github.com/moghbaie/NCBP-pipeline. The MS proteomics data have been deposited to the ProteomeXchange Consortium via the PRIDE (71) partner repository with the dataset identifier PXD016038. The protein interactions from this publication have been submitted to the IMEx (http://www.imexconsortium.org) consortium through IntAct (72) and assigned the identifier IM-27939.

## Supplementary Material

gkaa743_Supplemental_Files
