# Supplementary material for: Affinity proteomic dissection of the human nuclear cap-binding complex interactome
Source: Nucleic Acids Res. 2020 Sep 22;48(18):10456–69. doi: 10.1093/nar/gkaa743 (PMC7544204; doi:10.1093/nar/gkaa743)
Supplement: gkaa743_Supplemental_Files [file gkaa743_supplemental_files.zip › Supplemental_materials.zip!/Supplemental_materials (1)/GFP_normalization.pdf]

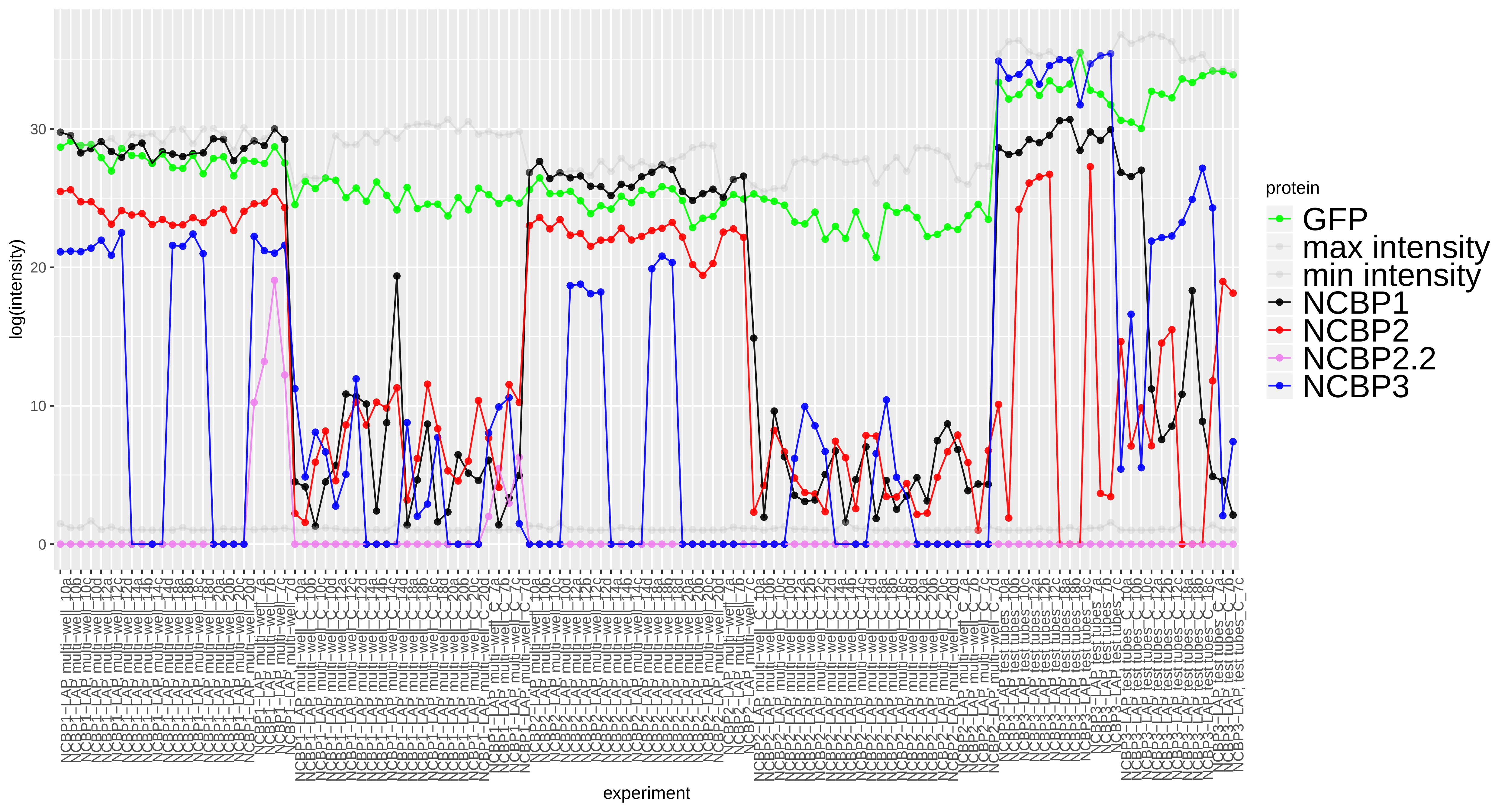
log(intensity)
0
10
20
30
experiment
protein
GFP
max intensity
min intensity
NCBP1
NCBP2
NCBP2.2
NCBP3

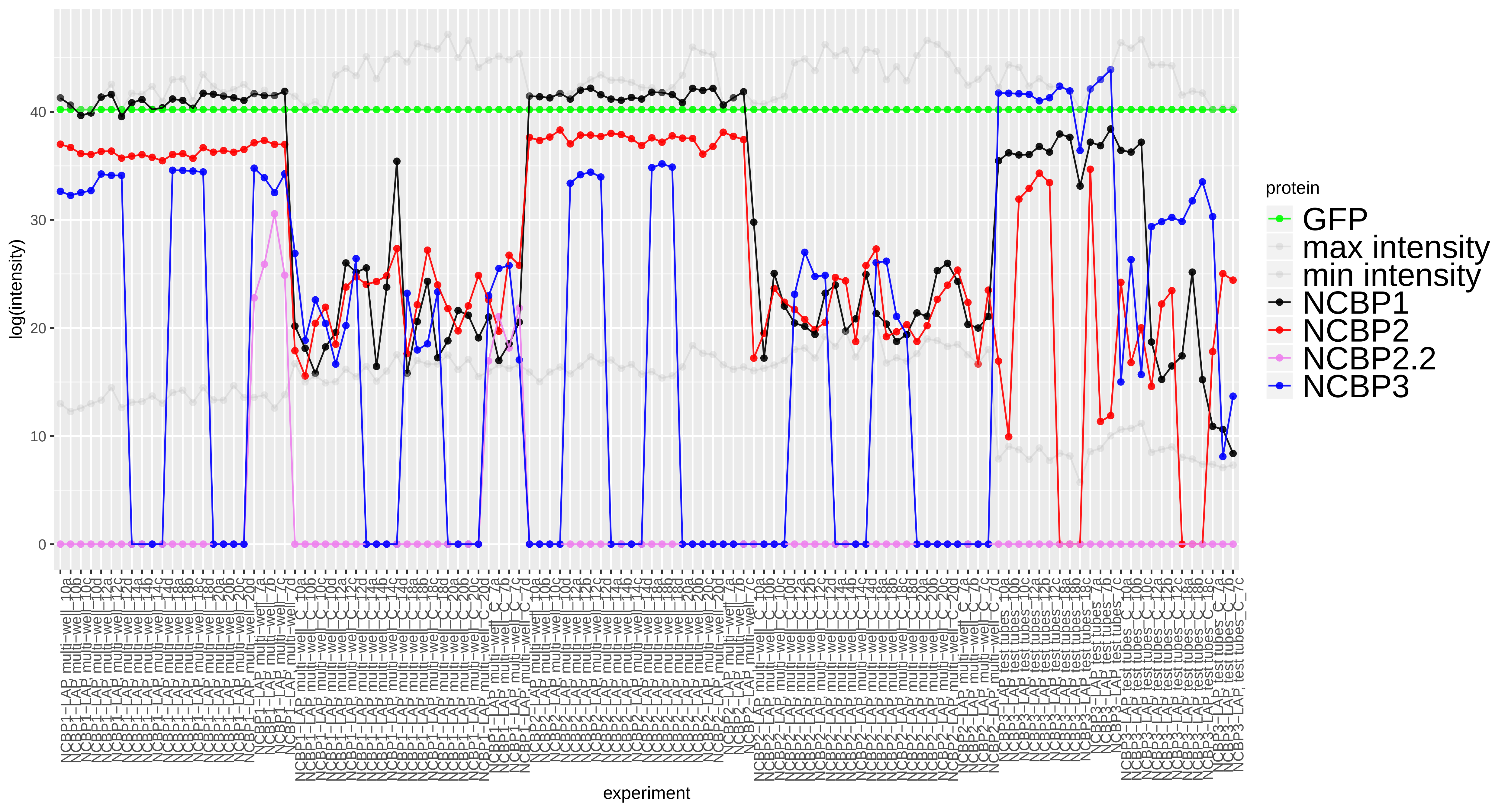

log(intensity)
40
30
20
10
0
experiment
protein
GFP
max intensity
min intensity
NCBP1
NCBP2
NCBP2.2
NCBP3
