# Supplementary material for: Affinity proteomic dissection of the human nuclear cap-binding complex interactome
Source: Nucleic Acids Res. 2020 Sep 22;48(18):10456–69. doi: 10.1093/nar/gkaa743 (PMC7544204; doi:10.1093/nar/gkaa743)
Supplement: gkaa743_Supplemental_Files [file gkaa743_supplemental_files.zip › Supplemental_materials.zip!/Supplemental_materials (1)/RMSE_NRMSE_plots.zip!/RMSE_combine/2019-06-14_20:03:26.conf.interval.rmse.with_iBAQ.pdf]

step4	RMSE
7	2.44±0.21
6	2.61±0.45
1	3.67±0.49
perseus	5.83±0.13
random	5.97±0.12
3	6.73±0.23
2	6.89±0.59
5	7.41±1.84
4	8.63±0.17
