# Supplementary material for: Affinity proteomic dissection of the human nuclear cap-binding complex interactome
Source: Nucleic Acids Res. 2020 Sep 22;48(18):10456–69. doi: 10.1093/nar/gkaa743 (PMC7544204; doi:10.1093/nar/gkaa743)
Supplement: gkaa743_Supplemental_Files [file gkaa743_supplemental_files.zip › Supplemental_materials.zip!/Supplemental_materials (1)/RMSE_NRMSE_plots.zip!/RMSE_combine/2019-06-08_23:15:31.conf.interval.rmse.with.pdf]

step4	RMSE
7	1.37±0.41
6	2.09±0.71
1	3.45±0.92
random	5±0.09
perseus	5.22±0.08
3	8.41±0.55
5	8.87±2.31
2	8.9±1.68
4	12.25±1.14
