# Supplementary material for: Affinity proteomic dissection of the human nuclear cap-binding complex interactome
Source: Nucleic Acids Res. 2020 Sep 22;48(18):10456–69. doi: 10.1093/nar/gkaa743 (PMC7544204; doi:10.1093/nar/gkaa743)
Supplement: gkaa743_Supplemental_Files [file gkaa743_supplemental_files.zip › Supplemental_materials.zip!/Supplemental_materials (1)/RMSE_NRMSE_plots.zip!/RMSE_combine/2019-06-14_22:43:06_nrmse_iBAQ.pdf]

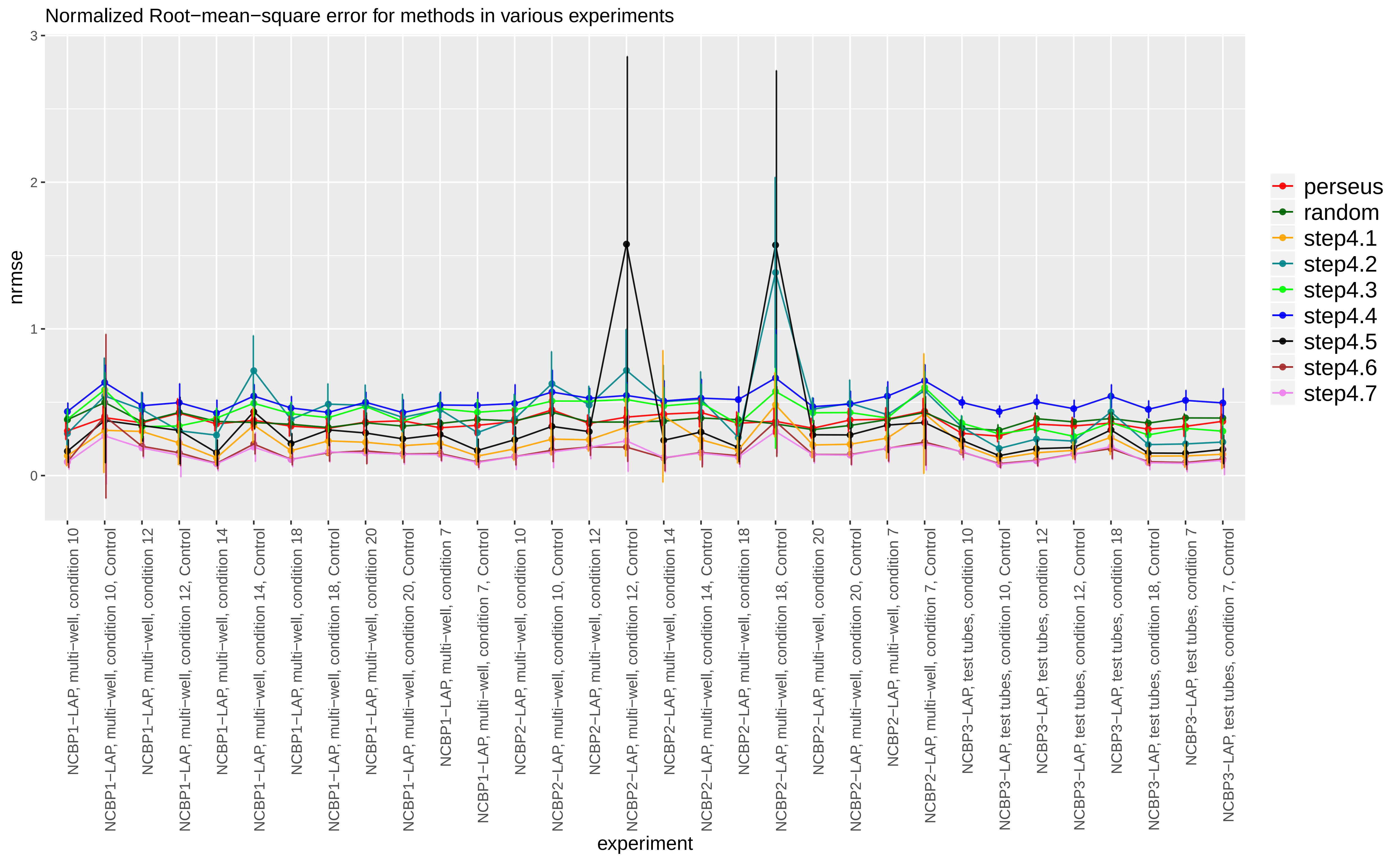

Normalized Root−mean−square error for methods in various experiments
nrmse
0
1
2
3
experiment
NCBP1−LAP, multi−well, condition 10
NCBP1−LAP, multi−well, condition 10, Control
NCBP1−LAP, multi−well, condition 12
NCBP1−LAP, multi−well, condition 12, Control
NCBP1−LAP, multi−well, condition 14
NCBP1−LAP, multi−well, condition 14, Control
NCBP1−LAP, multi−well, condition 18
NCBP1−LAP, multi−well, condition 18, Control
NCBP1−LAP, multi−well, condition 20
NCBP1−LAP, multi−well, condition 20, Control
NCBP1−LAP, multi−well, condition 7
NCBP1−LAP, multi−well, condition 7, Control
NCBP2−LAP, multi−well, condition 10
NCBP2−LAP, multi−well, condition 10, Control
NCBP2−LAP, multi−well, condition 12
NCBP2−LAP, multi−well, condition 12, Control
NCBP2−LAP, multi−well, condition 14
NCBP2−LAP, multi−well, condition 14, Control
NCBP2−LAP, multi−well, condition 18
NCBP2−LAP, multi−well, condition 18, Control
NCBP2−LAP, multi−well, condition 20
NCBP2−LAP, multi−well, condition 20, Control
NCBP2−LAP, multi−well, condition 7
NCBP2−LAP, multi−well, condition 7, Control
NCBP3−LAP, test tubes, condition 10
NCBP3−LAP, test tubes, condition 10, Control
NCBP3−LAP, test tubes, condition 12
NCBP3−LAP, test tubes, condition 12, Control
NCBP3−LAP, test tubes, condition 18
NCBP3−LAP, test tubes, condition 18, Control
NCBP3−LAP, test tubes, condition 7
NCBP3−LAP, test tubes, condition 7, Control
perseus
random
step4.1
step4.2
step4.3
step4.4
step4.5
step4.6
step4.7
