# Supplementary material for: Affinity proteomic dissection of the human nuclear cap-binding complex interactome
Source: Nucleic Acids Res. 2020 Sep 22;48(18):10456–69. doi: 10.1093/nar/gkaa743 (PMC7544204; doi:10.1093/nar/gkaa743)

NCBP1−LAP, multi−well, condition 7

cases

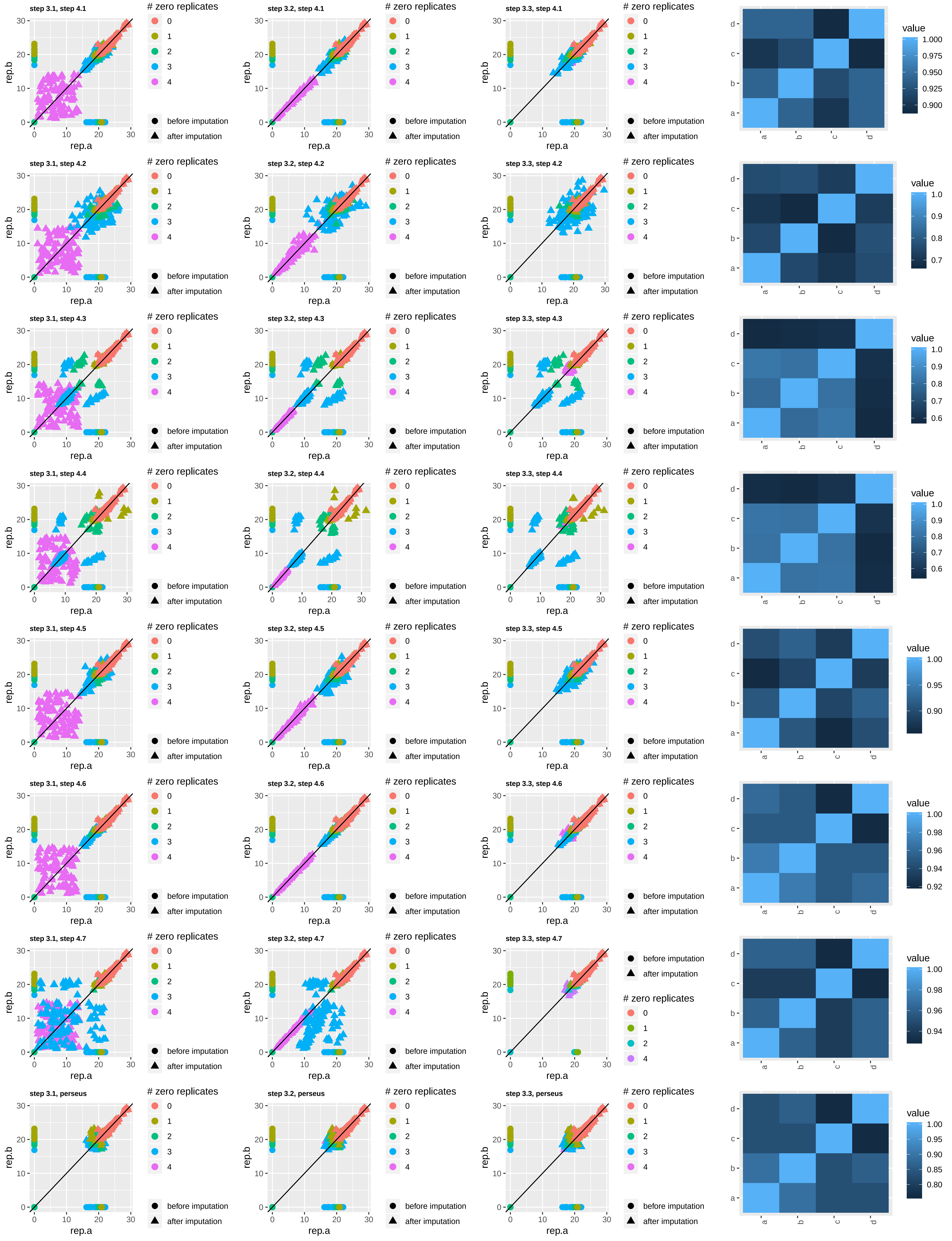

NCBP1−LAP, multi−well, condition 7

controls

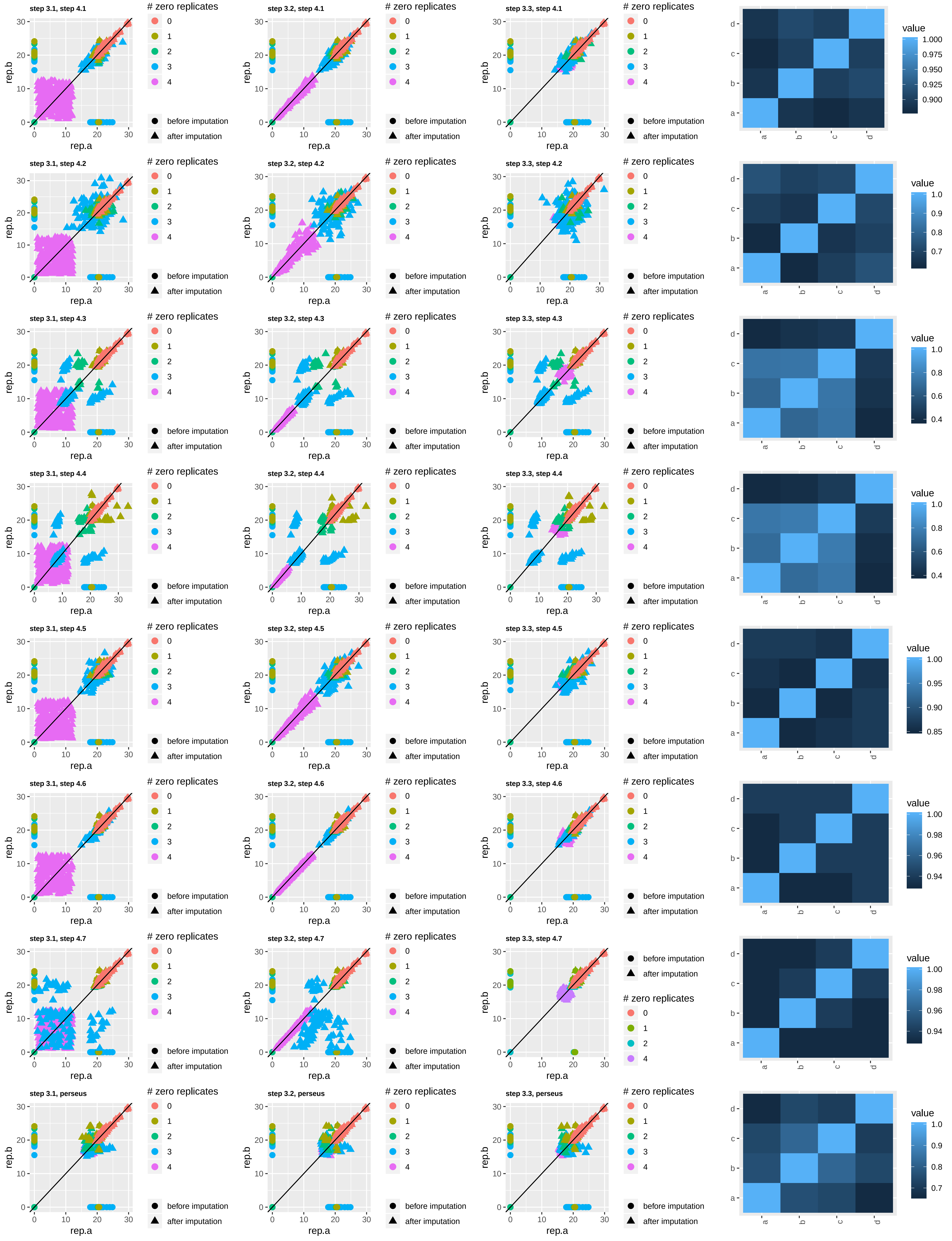

NCBP1−LAP, multi-well, condition 10

cases

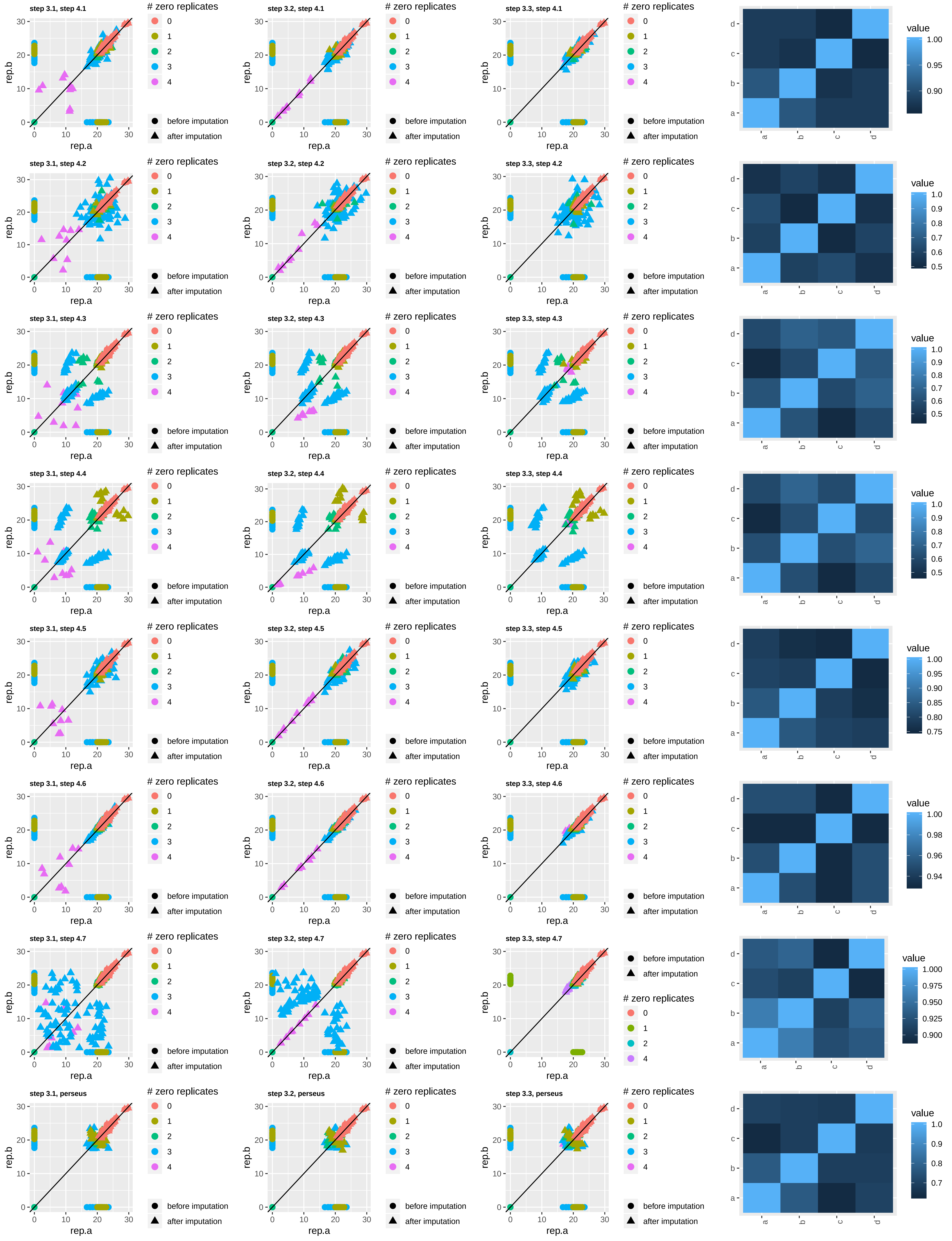

NCBP1−LAP, multi-well, condition 10

controls

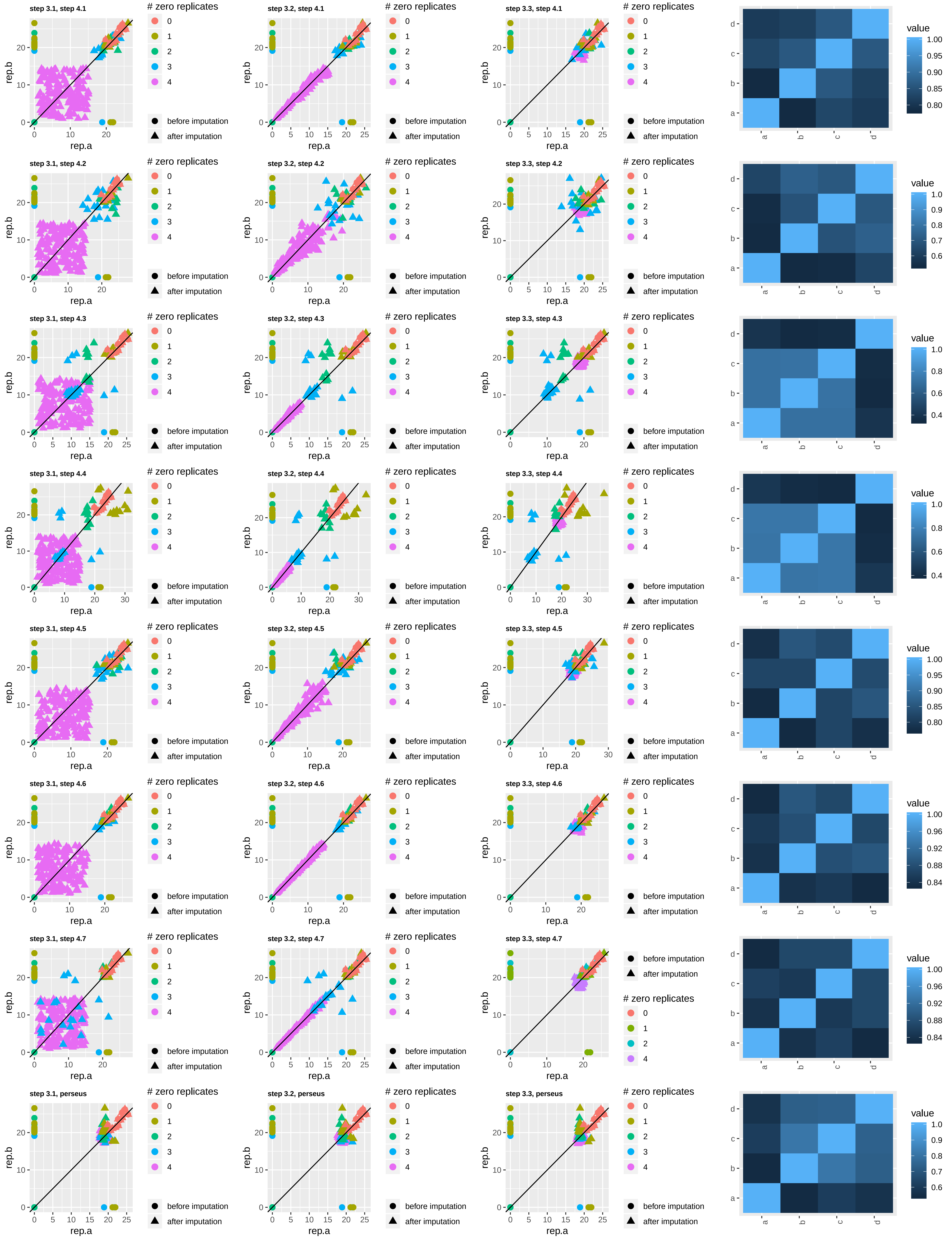

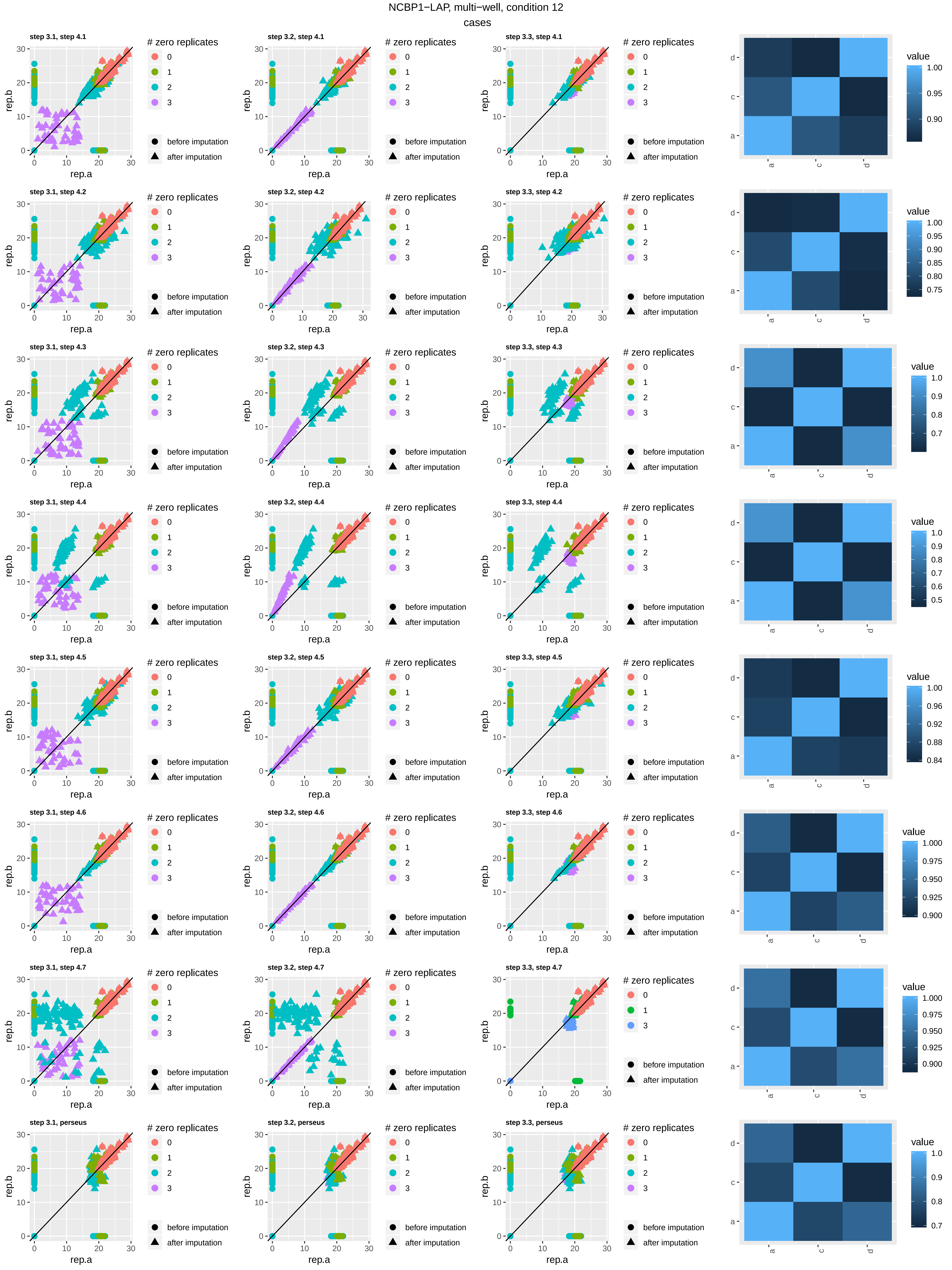

NCBP1−LAP, multi-well, condition 12
cases
step 3.1, step 4.1
step 3.2, step 4.1
step 3.3, step 4.1
step 3.1, step 4.2
step 3.2, step 4.2
step 3.3, step 4.2
step 3.1, step 4.3
step 3.2, step 4.3
step 3.3, step 4.3
step 3.1, step 4.4
step 3.2, step 4.4
step 3.3, step 4.4
step 3.1, step 4.5
step 3.2, step 4.5
step 3.3, step 4.5
step 3.1, step 4.6
step 3.2, step 4.6
step 3.3, step 4.6
step 3.1, step 4.7
step 3.2, step 4.7
step 3.3, step 4.7
step 3.1, perseus
step 3.2, perseus
step 3.3, perseus
rep.a
rep.b
zero replicates
0
1
2
3
before imputation
after imputation
value
a
c
d

NCBP1−LAP, multi−well, condition 12
controls

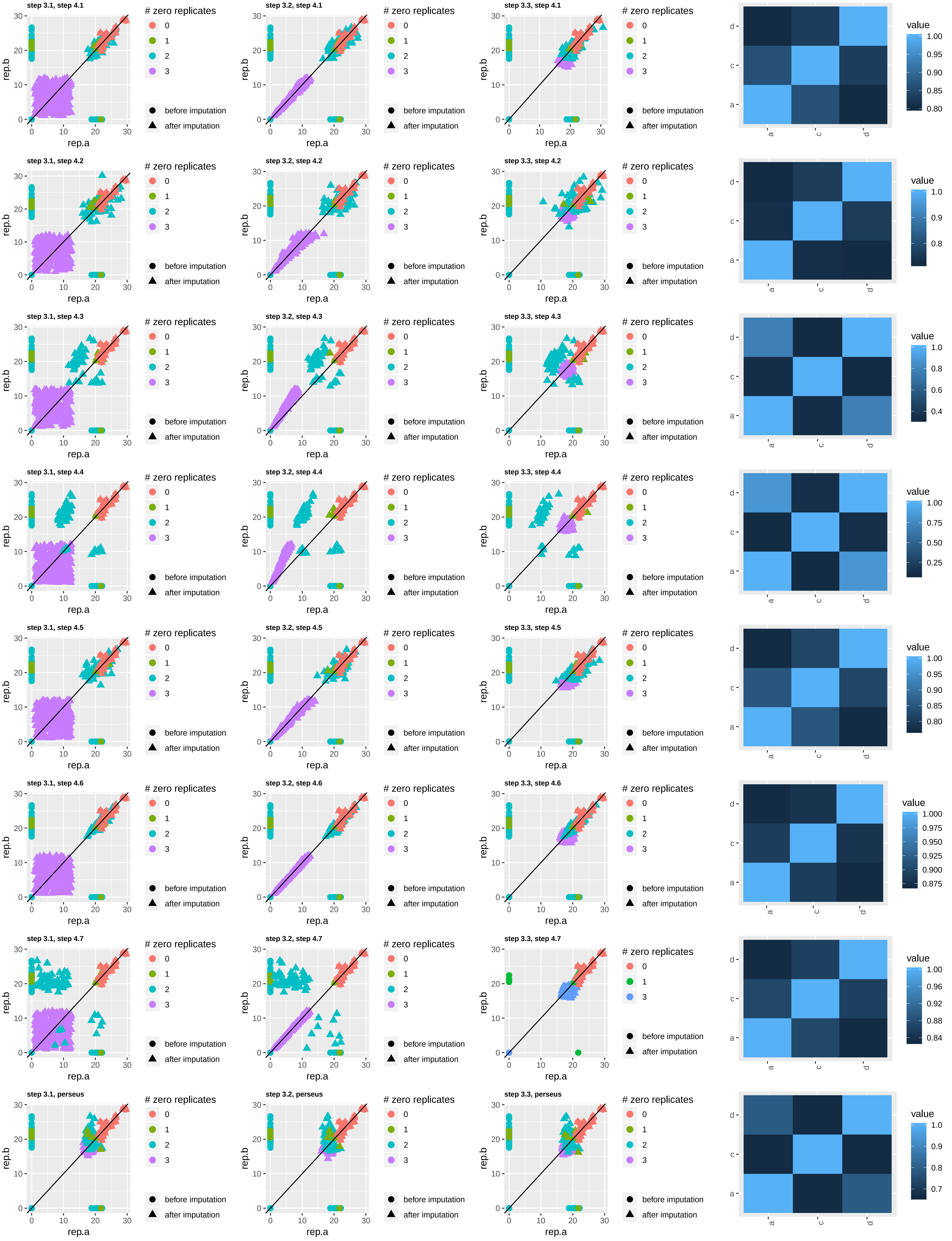

NCBP1−LAP, multi−well, condition 14
cases

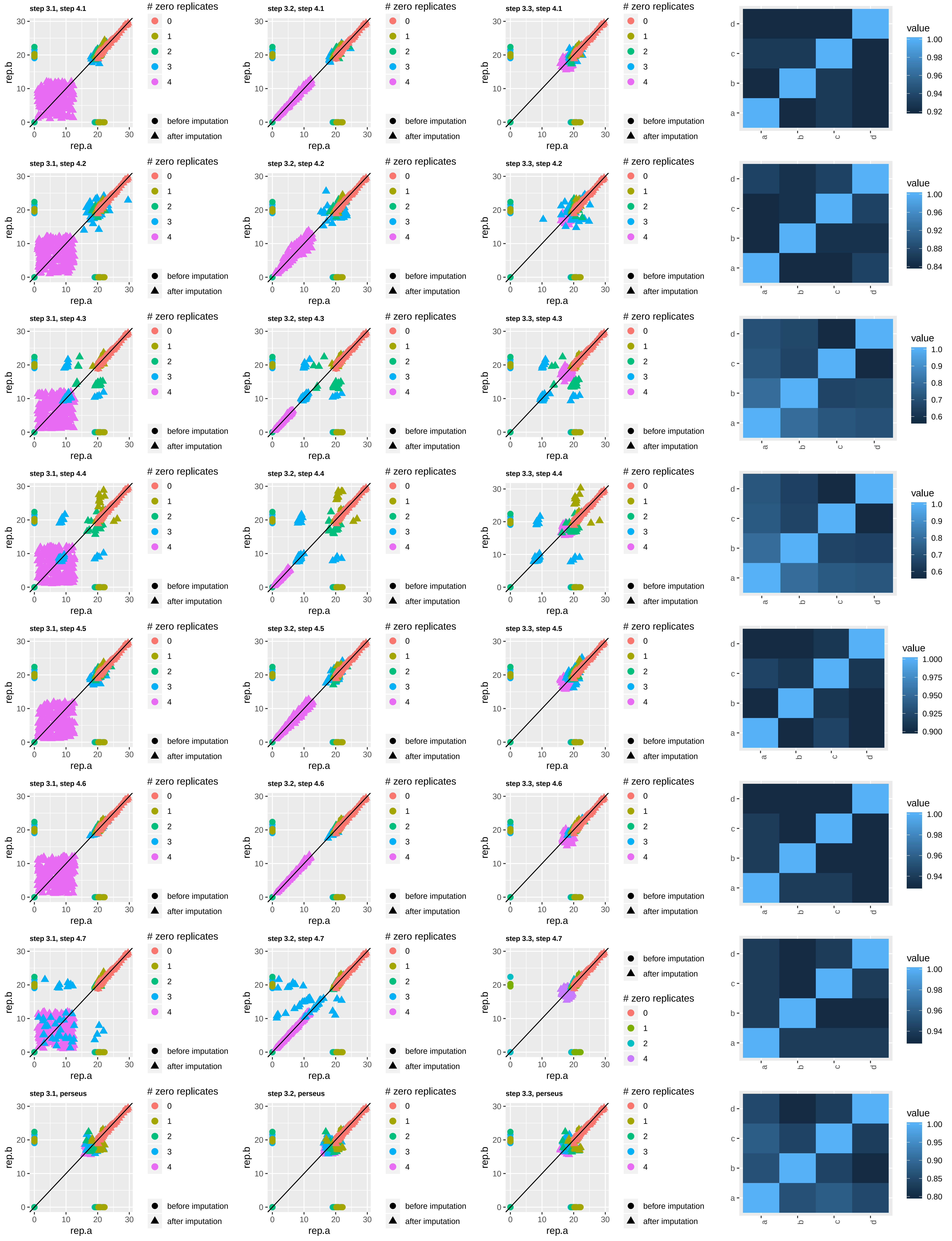

NCBP1−LAP, multi-well, condition 14

controls

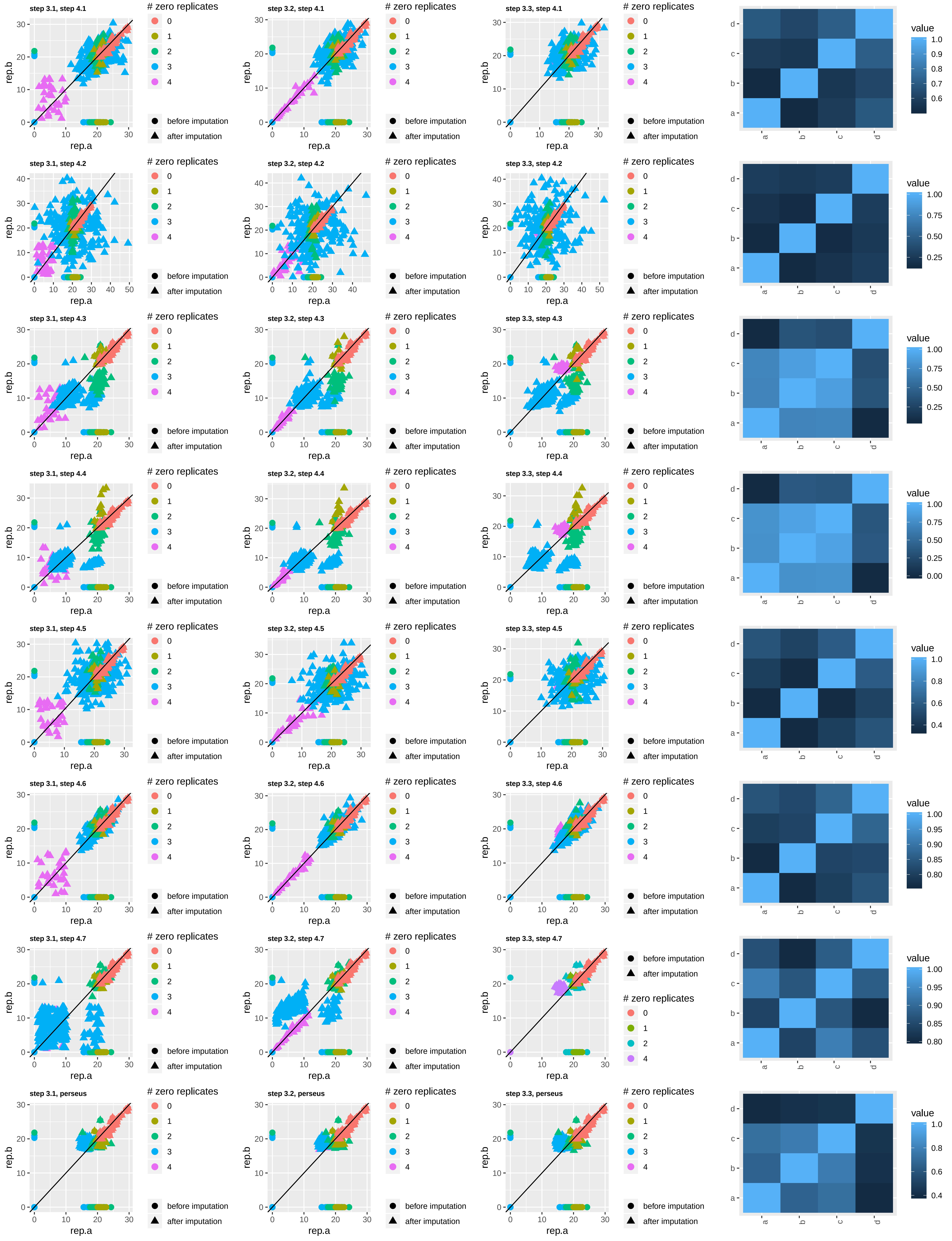

NCBP1−LAP, multi−well, condition 18

cases

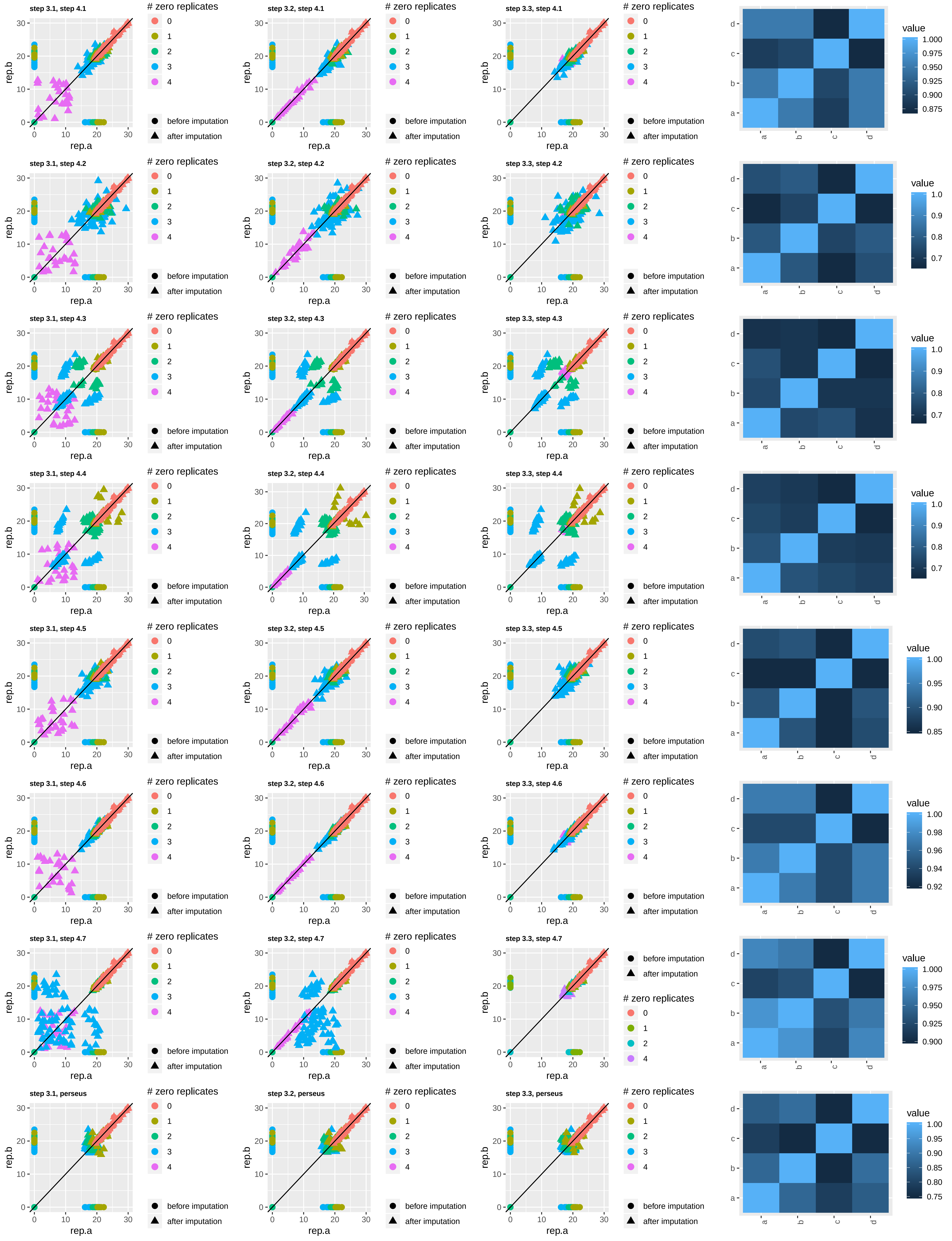

NCBP1−LAP, multi−well, condition 18

controls

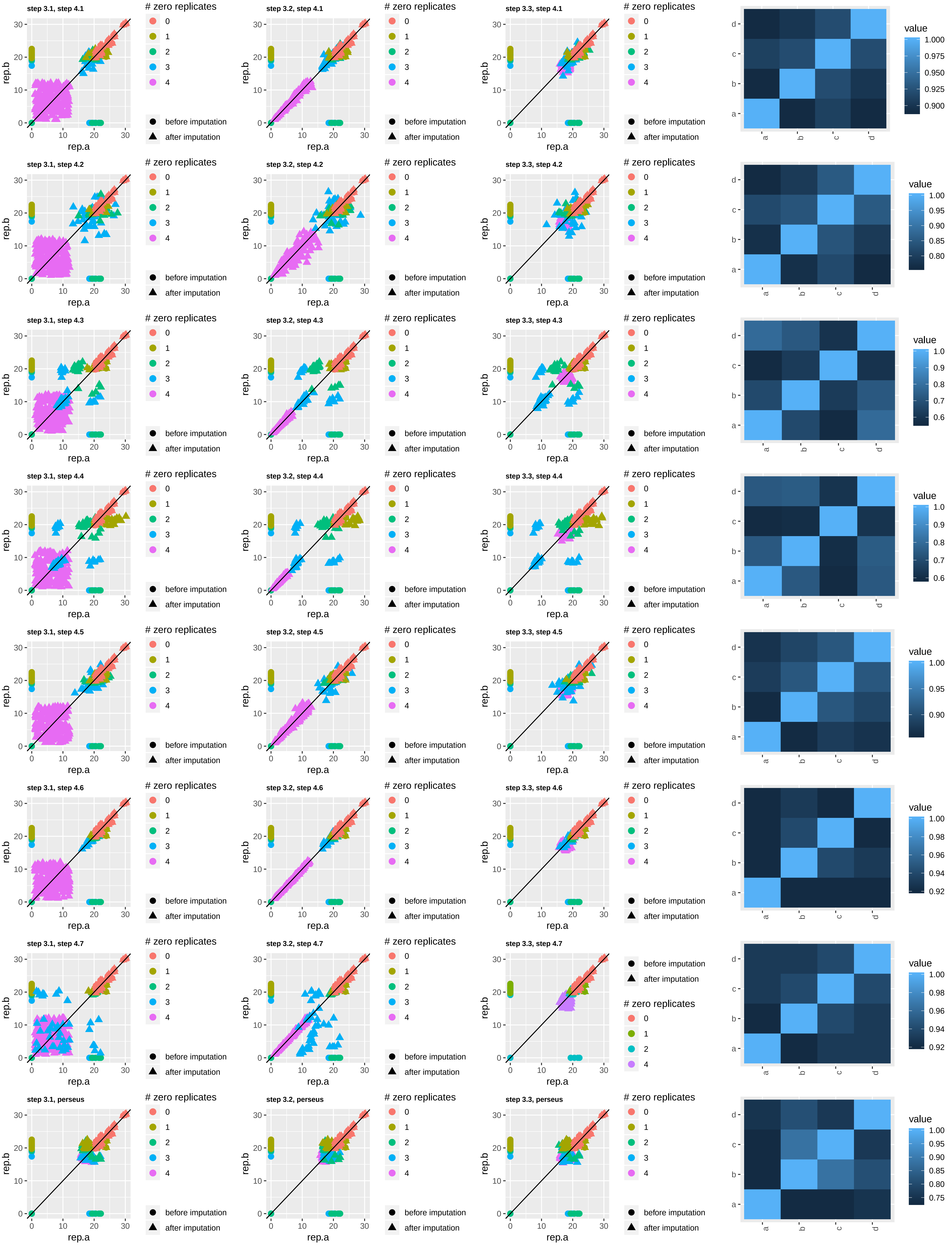

NCBP1−LAP, multi-well, condition 20 cases

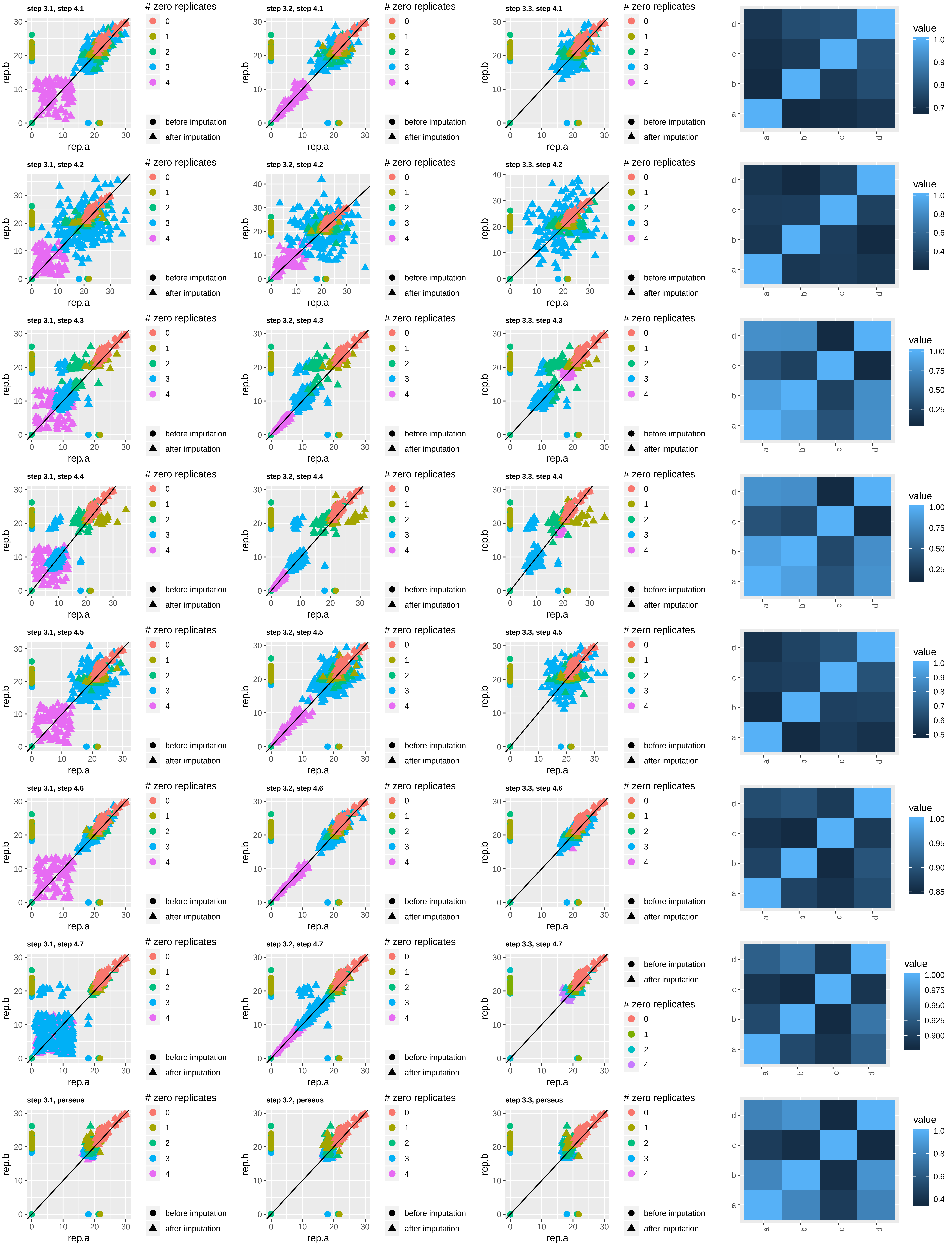

NCBP1−LAP, multi-well, condition 20

controls

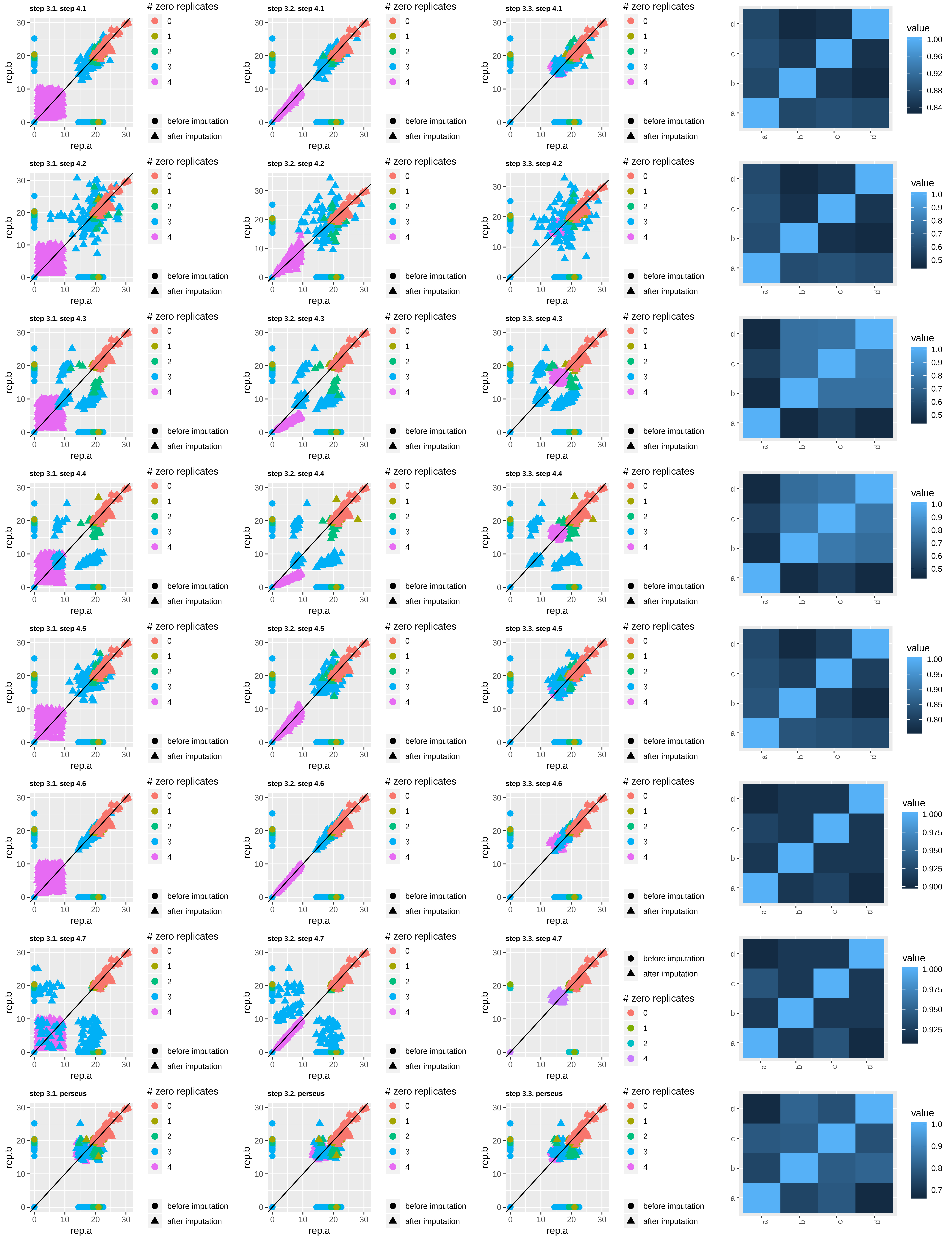

NCBP2−LAP, multi−well, condition 7

cases

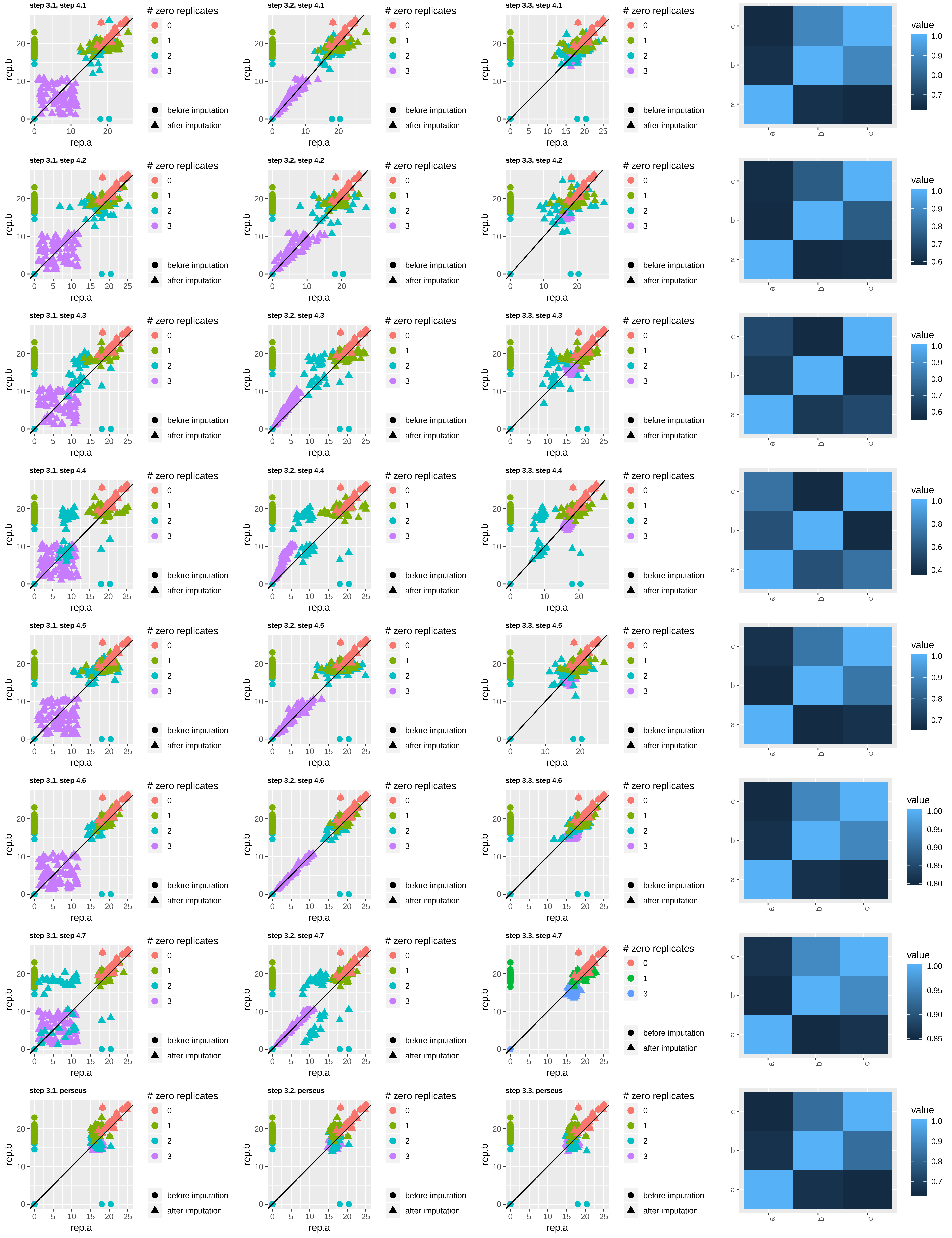

NCBP2−LAP, multi−well, condition 7

controls

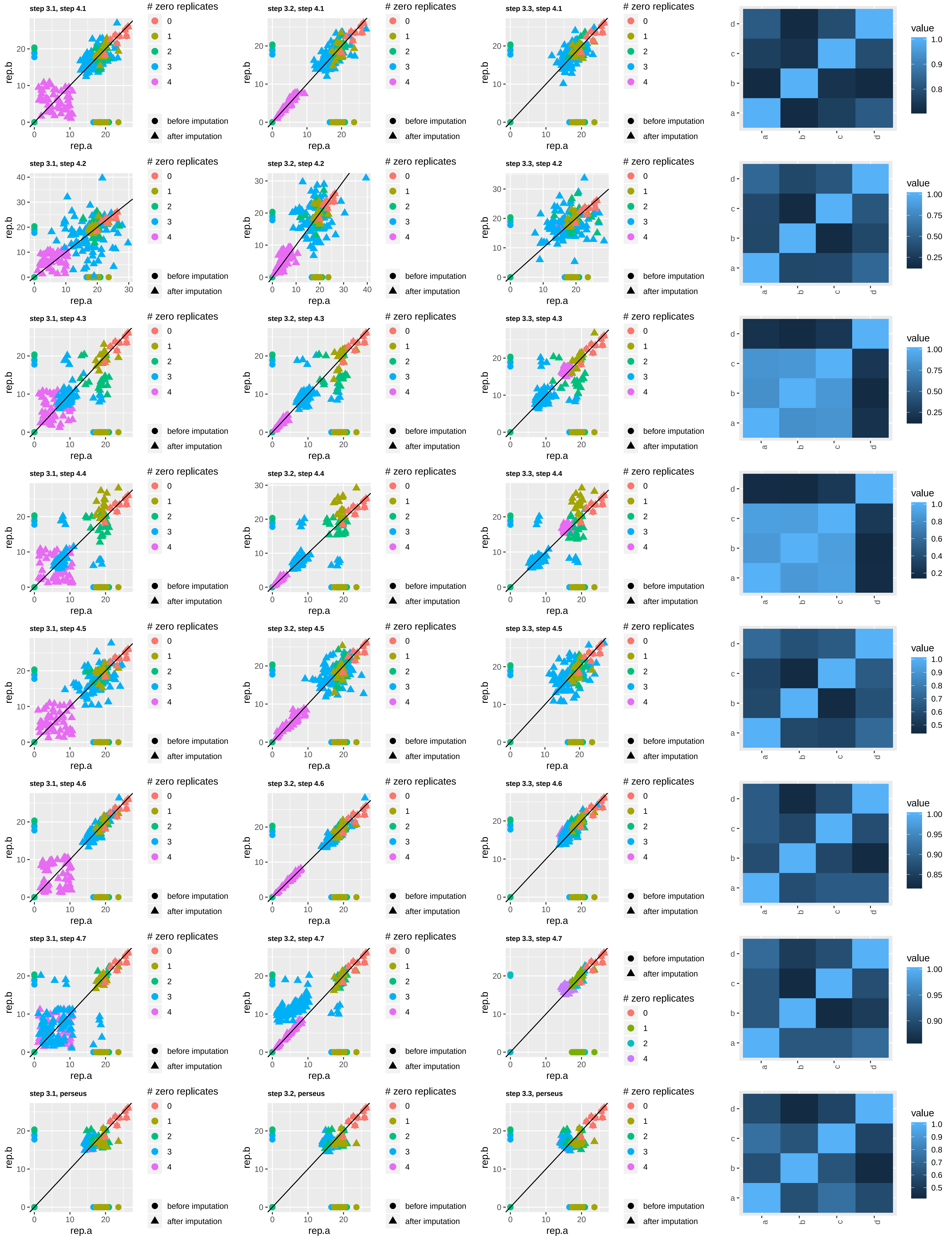

NCBP2−LAP, multi−well, condition 10
cases

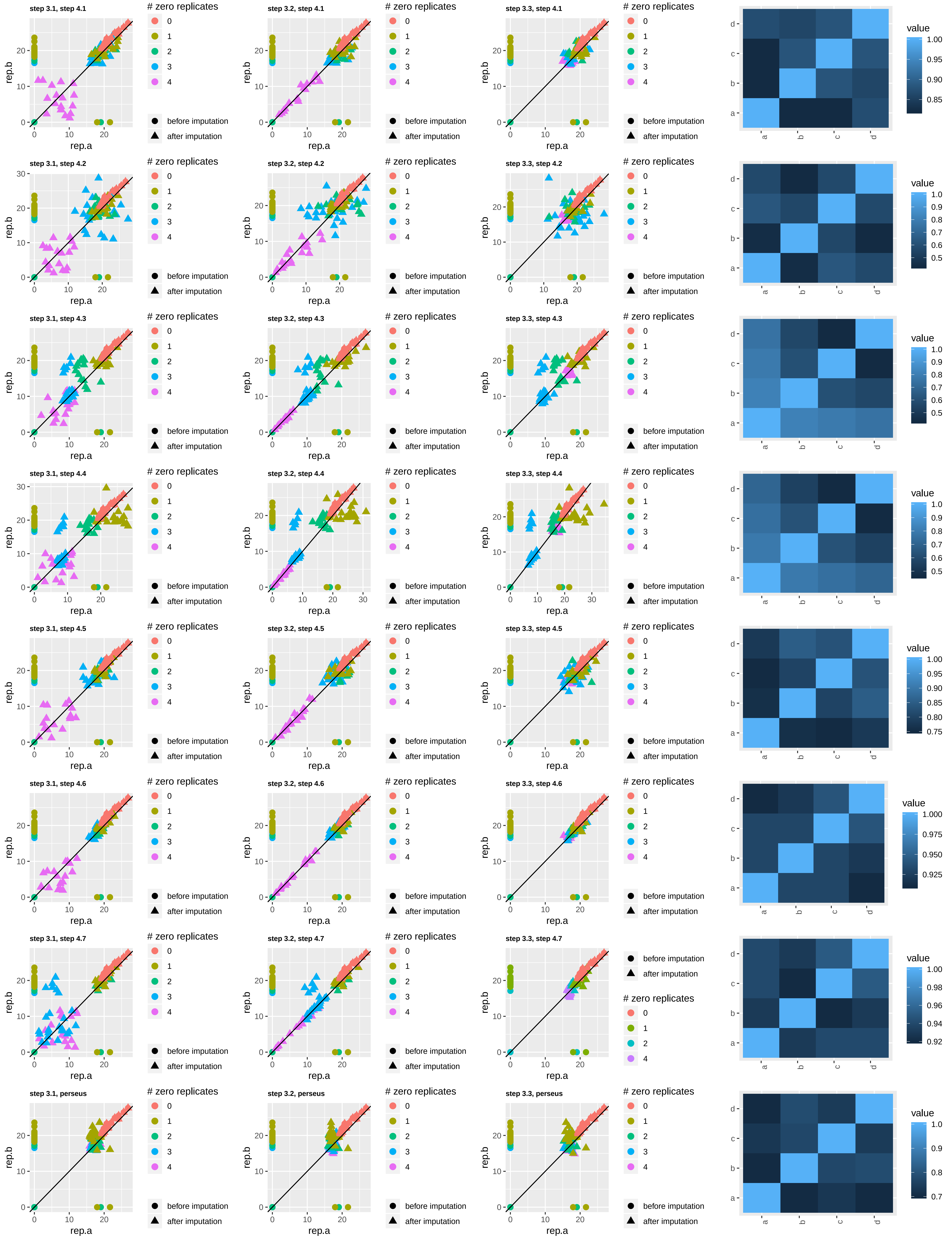

NCBP2−LAP, multi-well, condition 10

controls

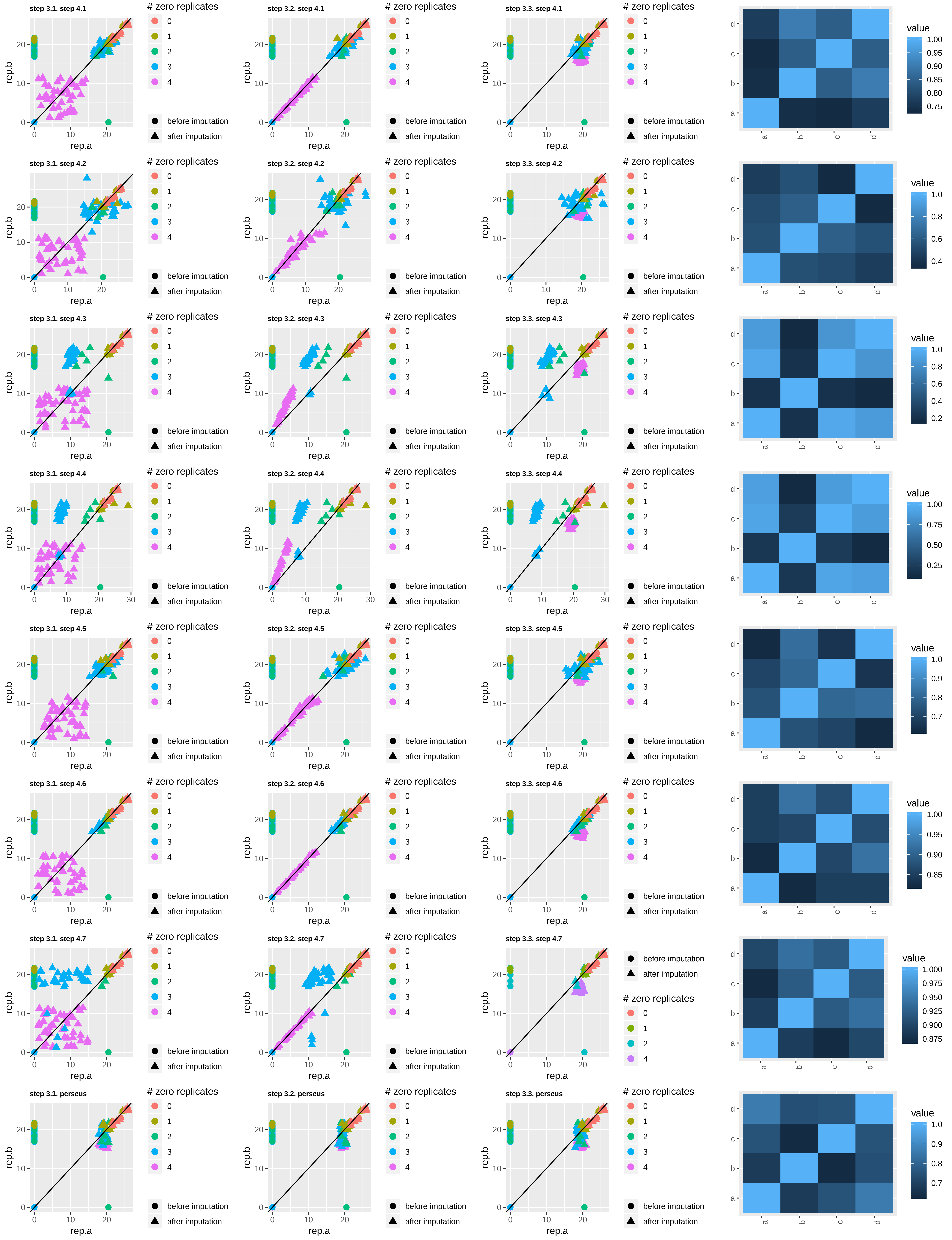

NCBP2−LAP, multi-well, condition 12 cases

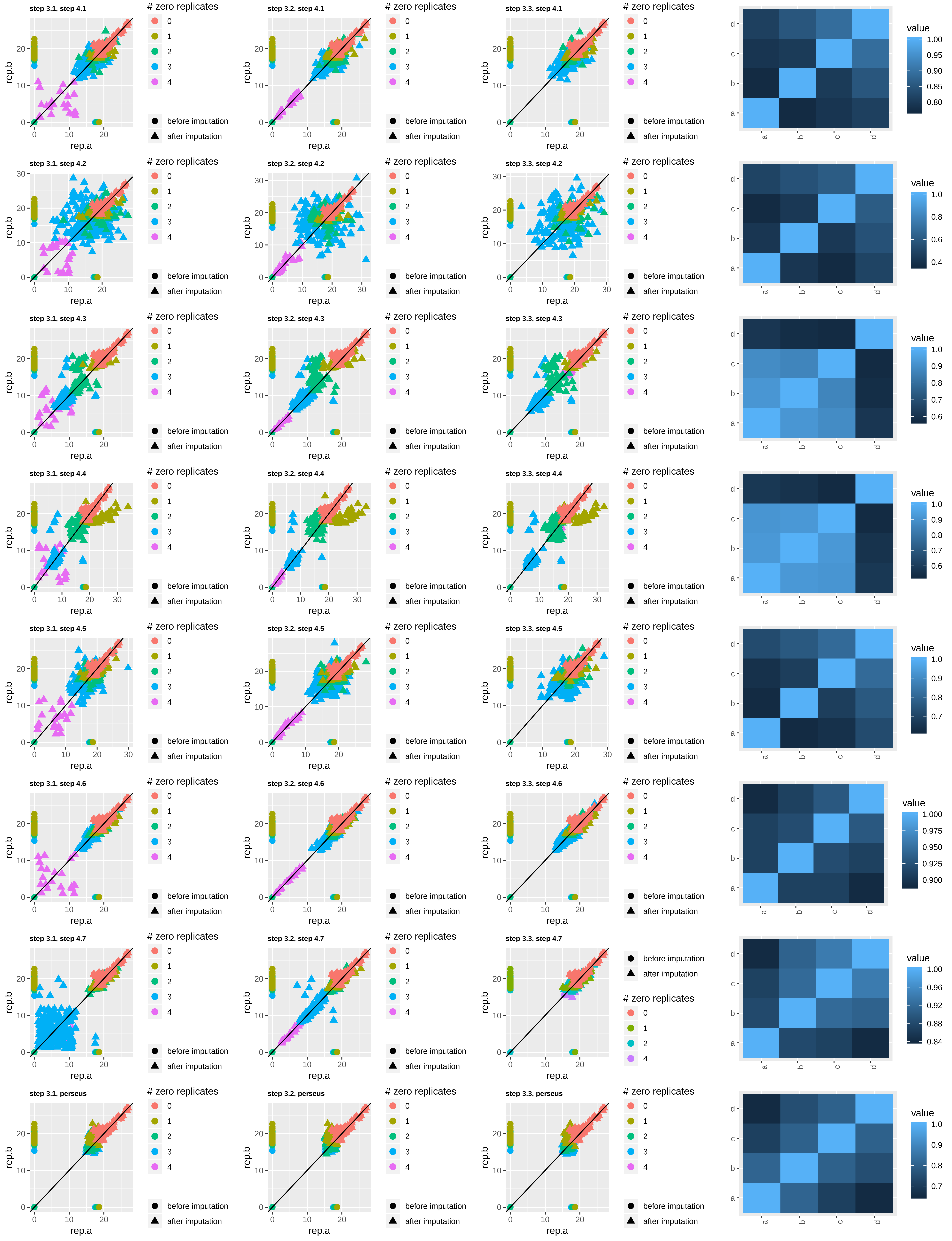

NCBP2−LAP, multi−well, condition 12

controls

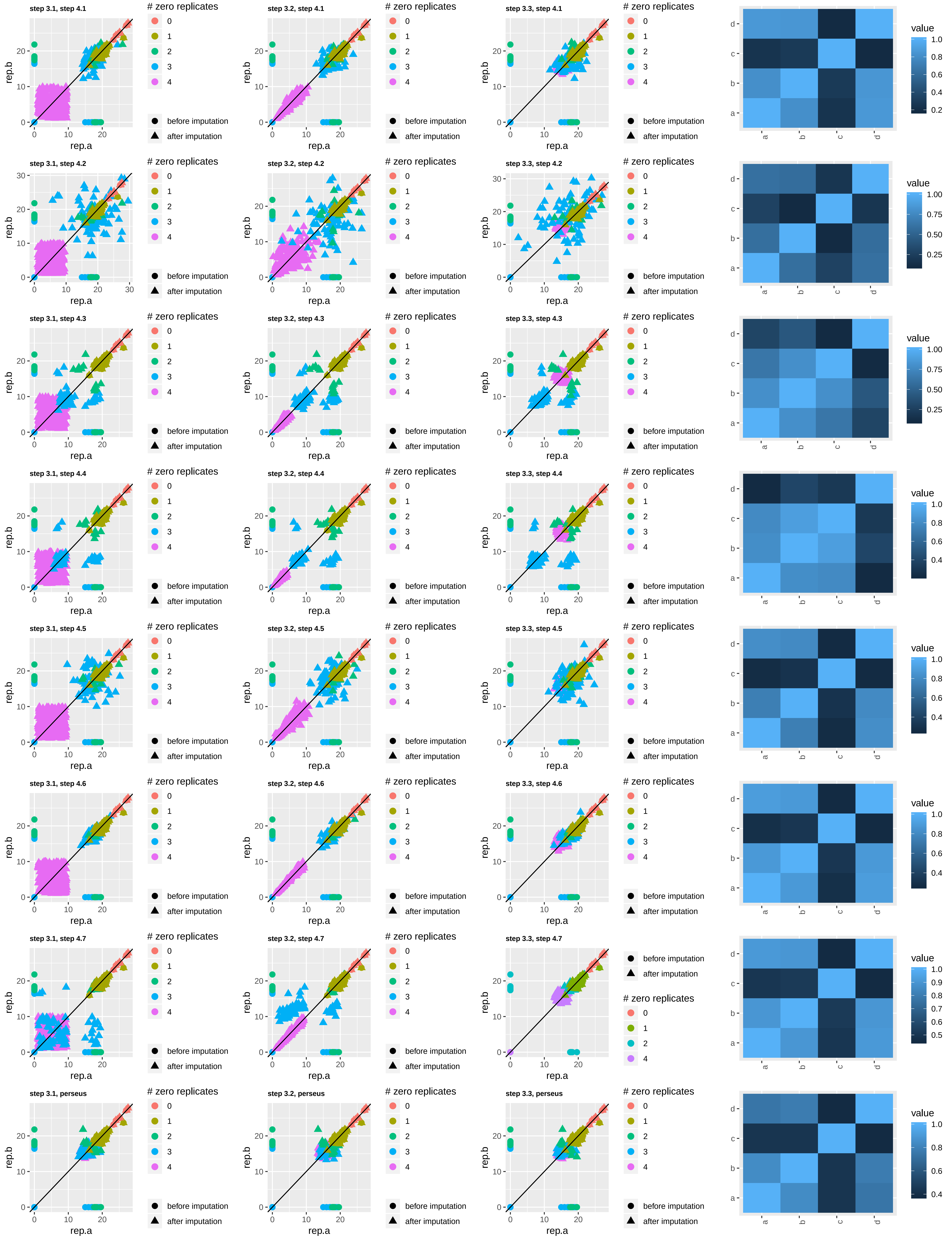

NCBP2−LAP, multi−well, condition 14

cases

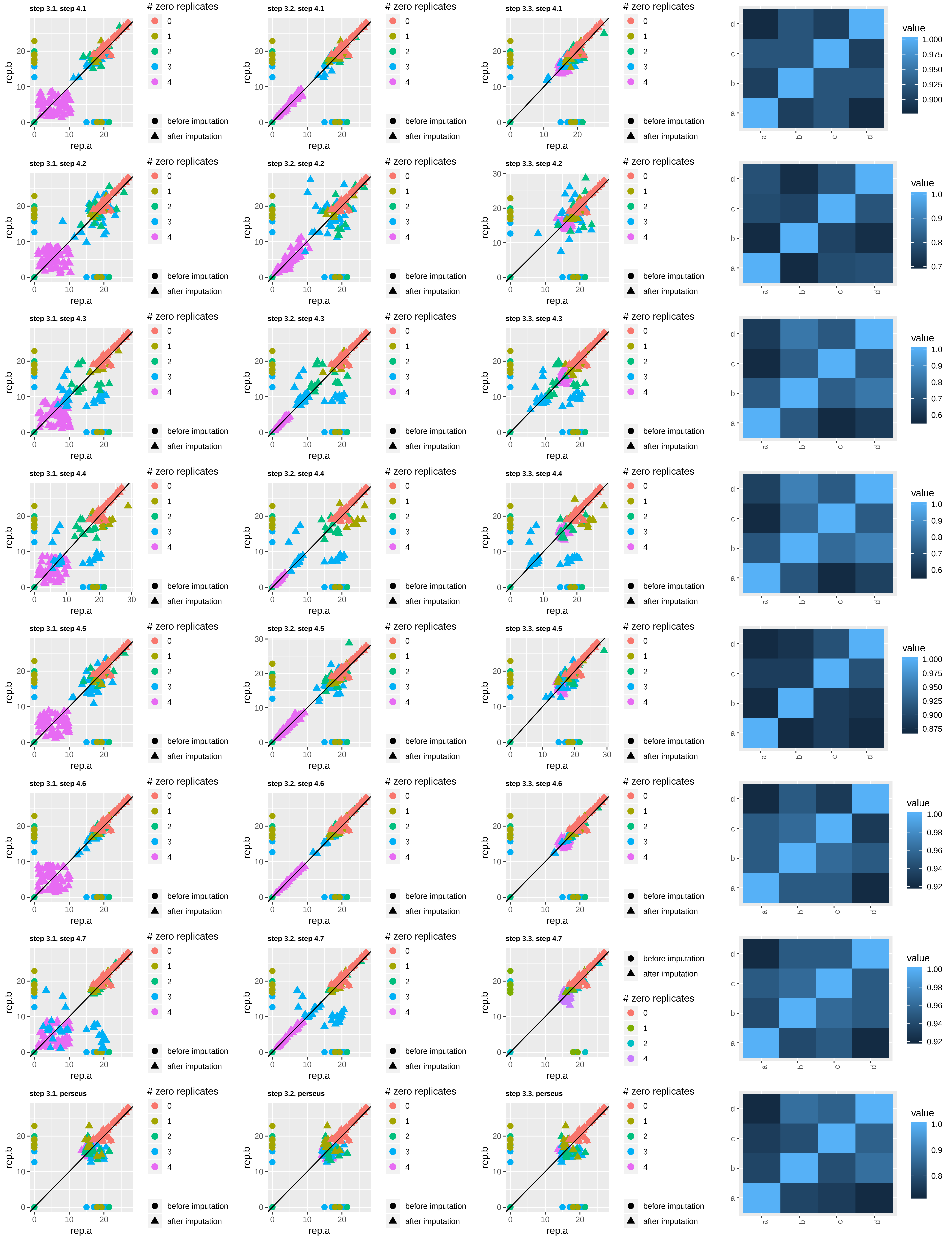

NCBP2−LAP, multi−well, condition 14

controls

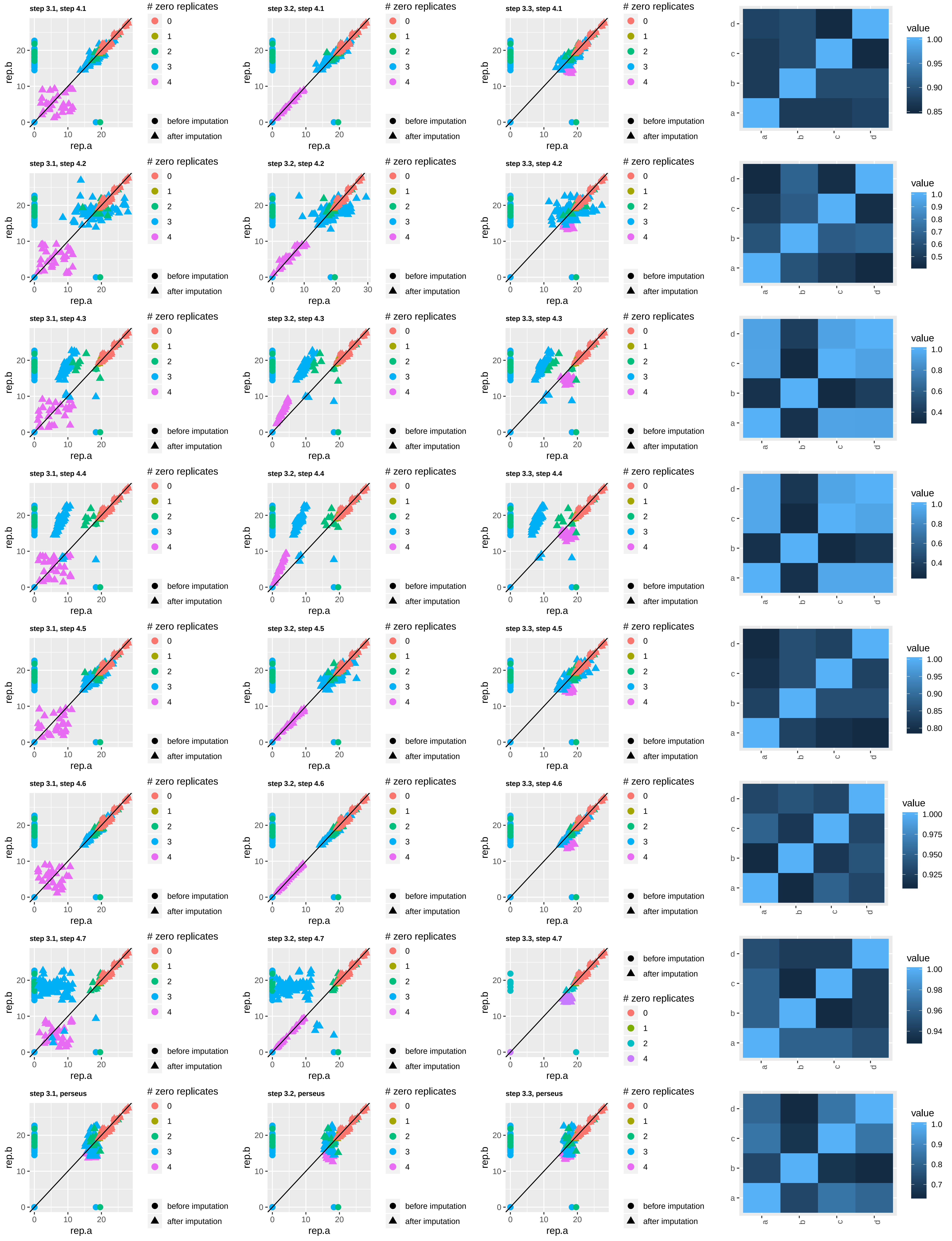

NCBP2−LAP, multi−well, condition 18

cases

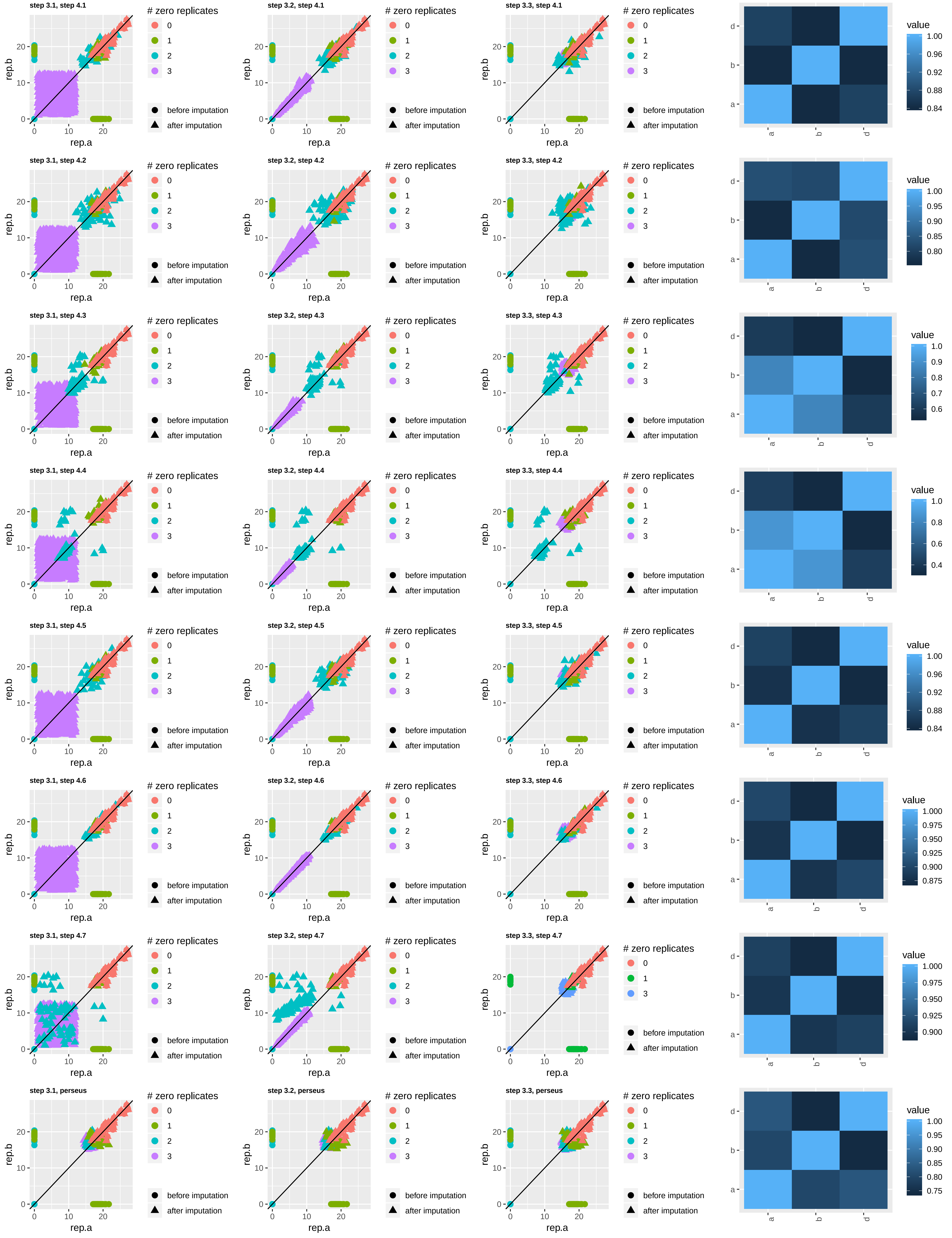

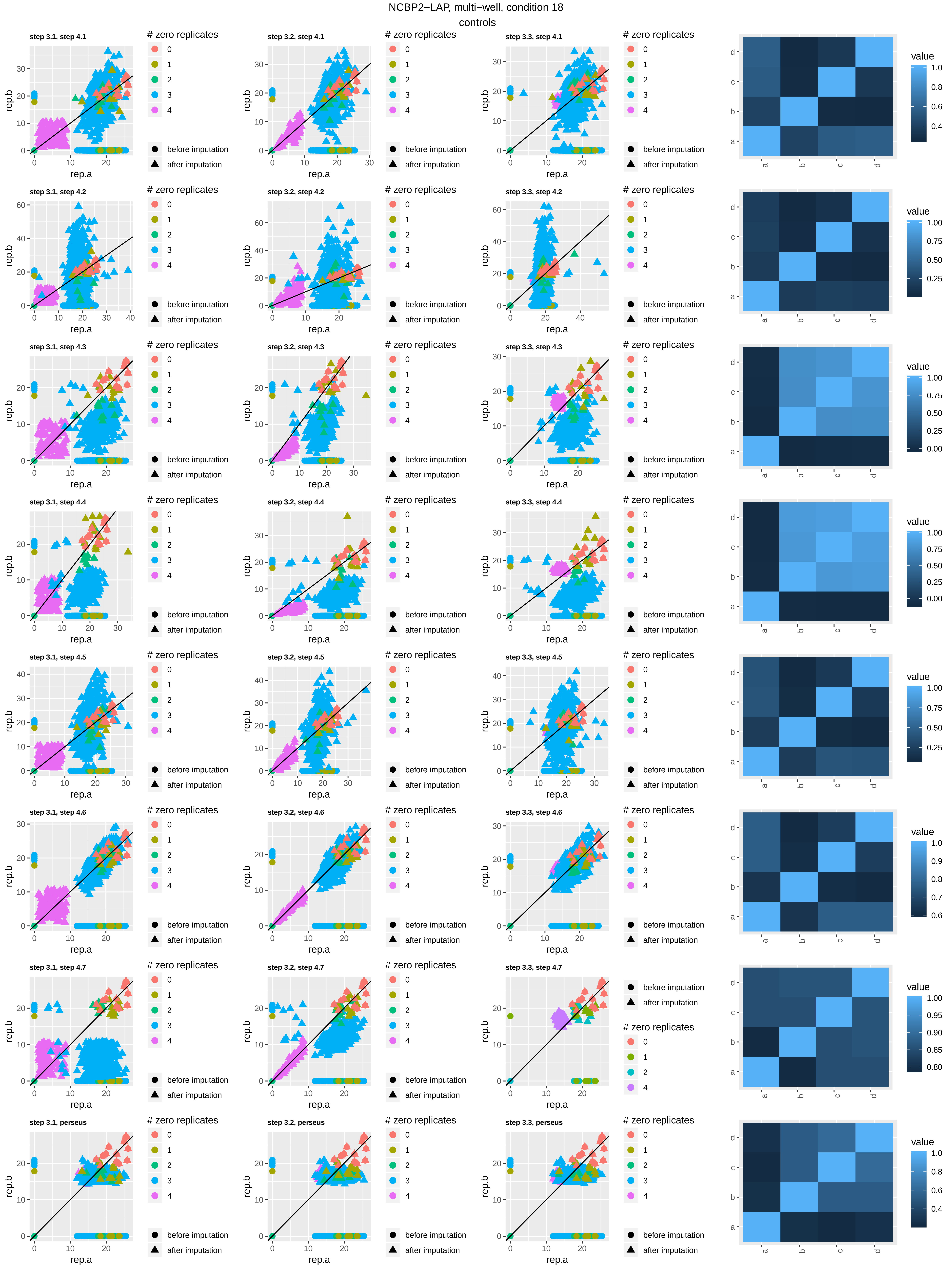

NCBP2−LAP, multi−well, condition 18
controls
step 3.1, step 4.1
step 3.2, step 4.1
step 3.3, step 4.1
step 3.1, step 4.2
step 3.2, step 4.2
step 3.3, step 4.2
step 3.1, step 4.3
step 3.2, step 4.3
step 3.3, step 4.3
step 3.1, step 4.4
step 3.2, step 4.4
step 3.3, step 4.4
step 3.1, step 4.5
step 3.2, step 4.5
step 3.3, step 4.5
step 3.1, step 4.6
step 3.2, step 4.6
step 3.3, step 4.6
step 3.1, step 4.7
step 3.2, step 4.7
step 3.3, step 4.7
step 3.1, perseus
step 3.2, perseus
step 3.3, perseus
rep.a
rep.b
zero replicates
0
1
2
3
4
before imputation
after imputation
value
a
b
c
d

NCBP2−LAP, multi−well, condition 20

cases

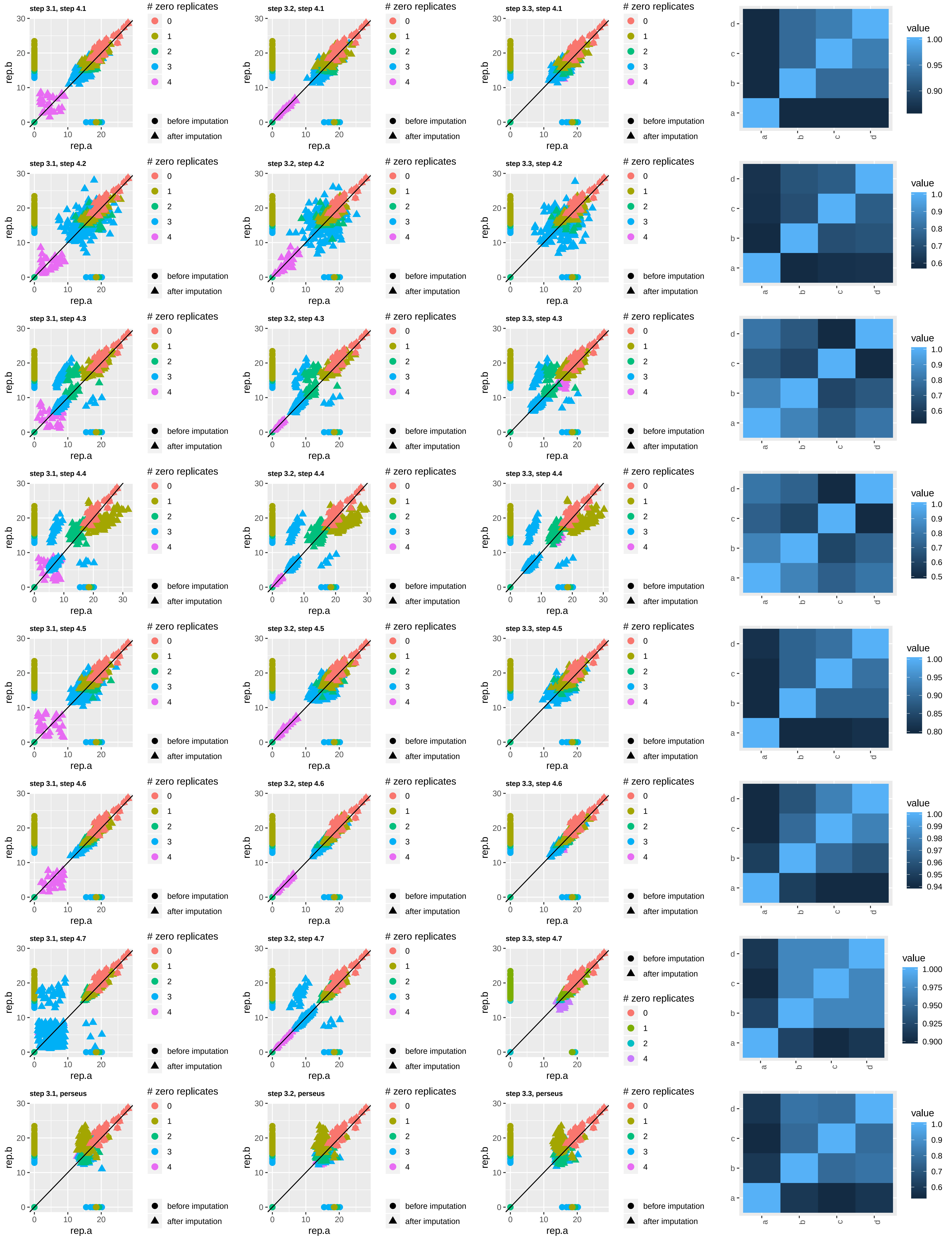

NCBP2−LAP, multi−well, condition 20

controls

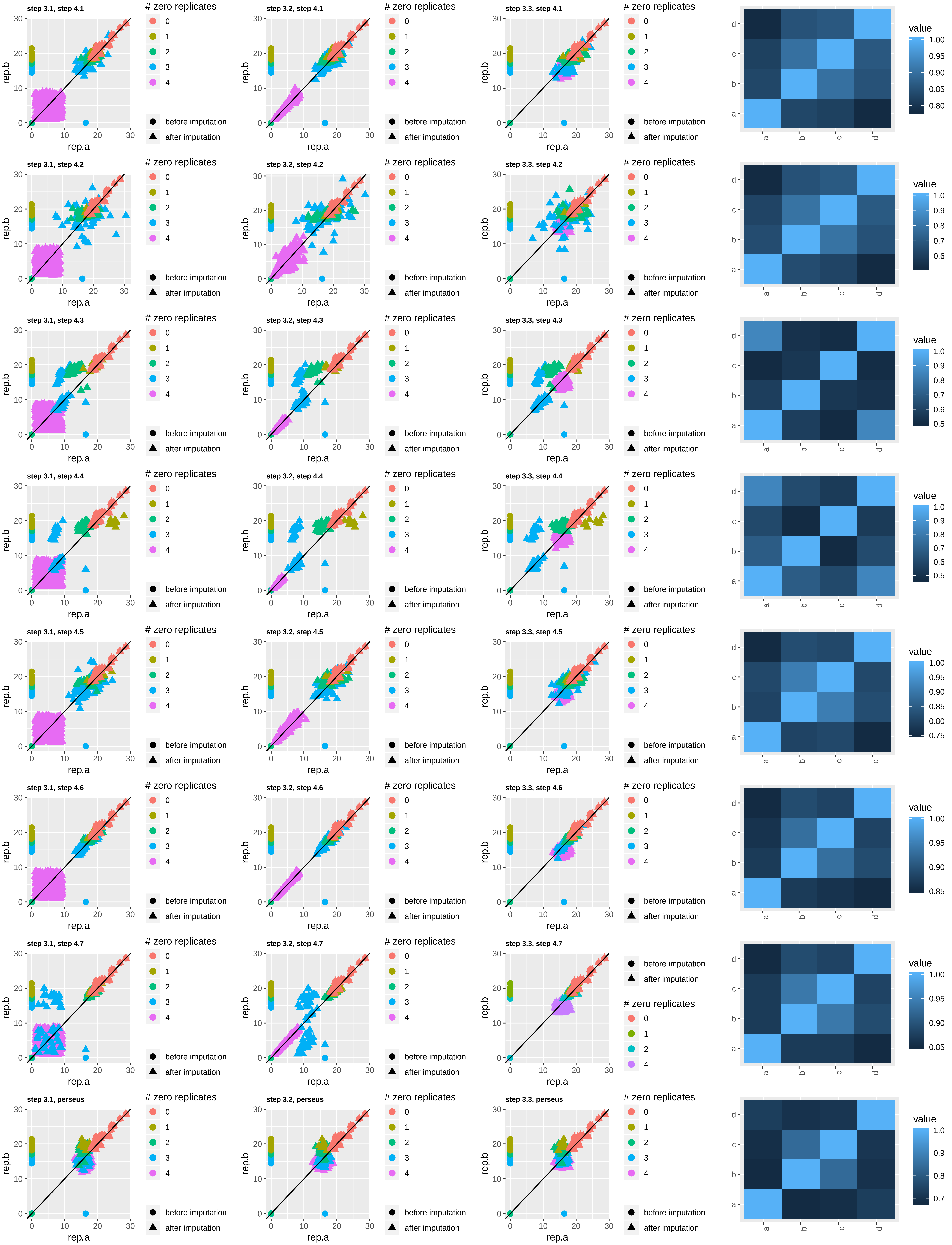

NCBP3−LAP, test tubes, condition 7
cases

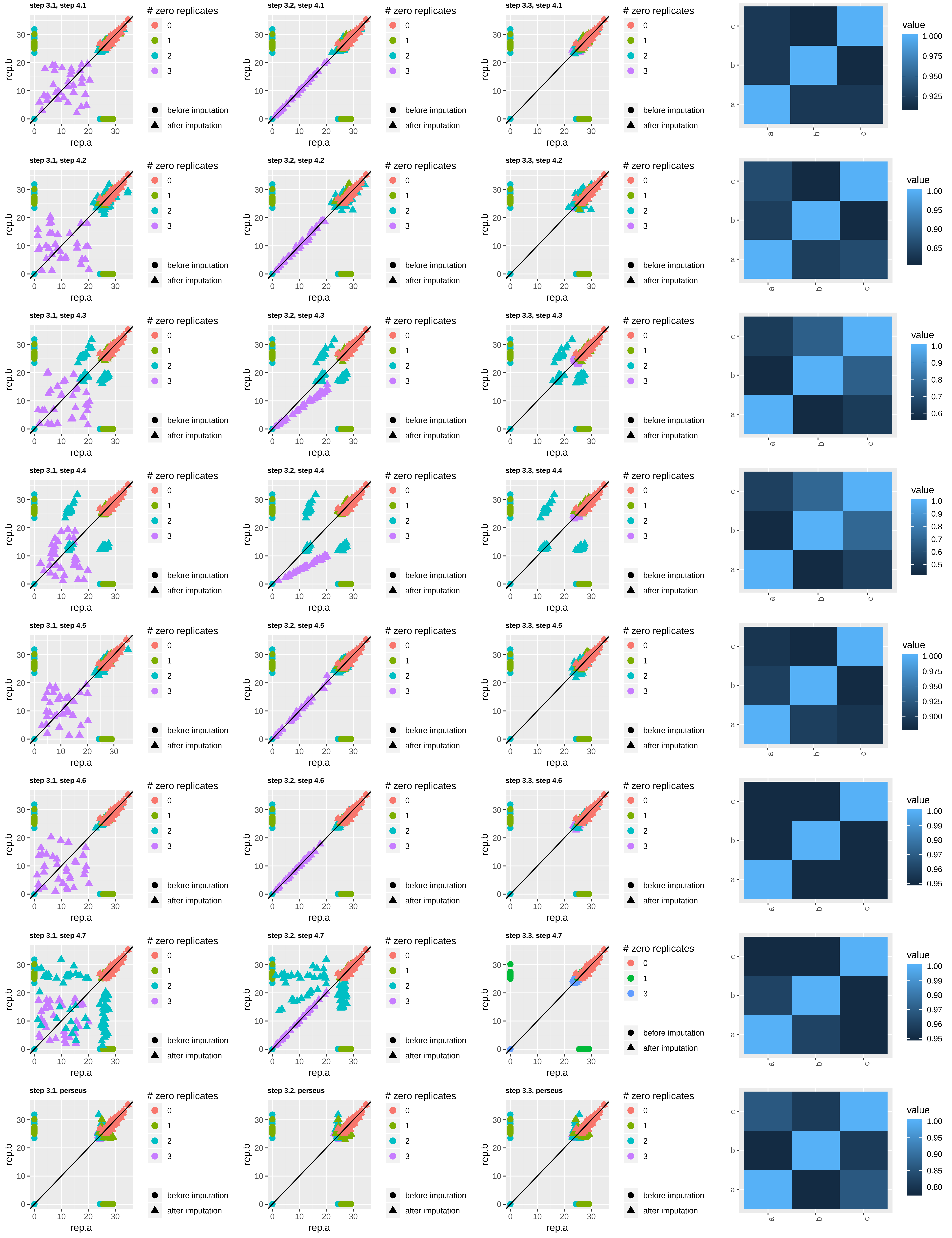

NCBP3−LAP, test tubes, condition 7

controls

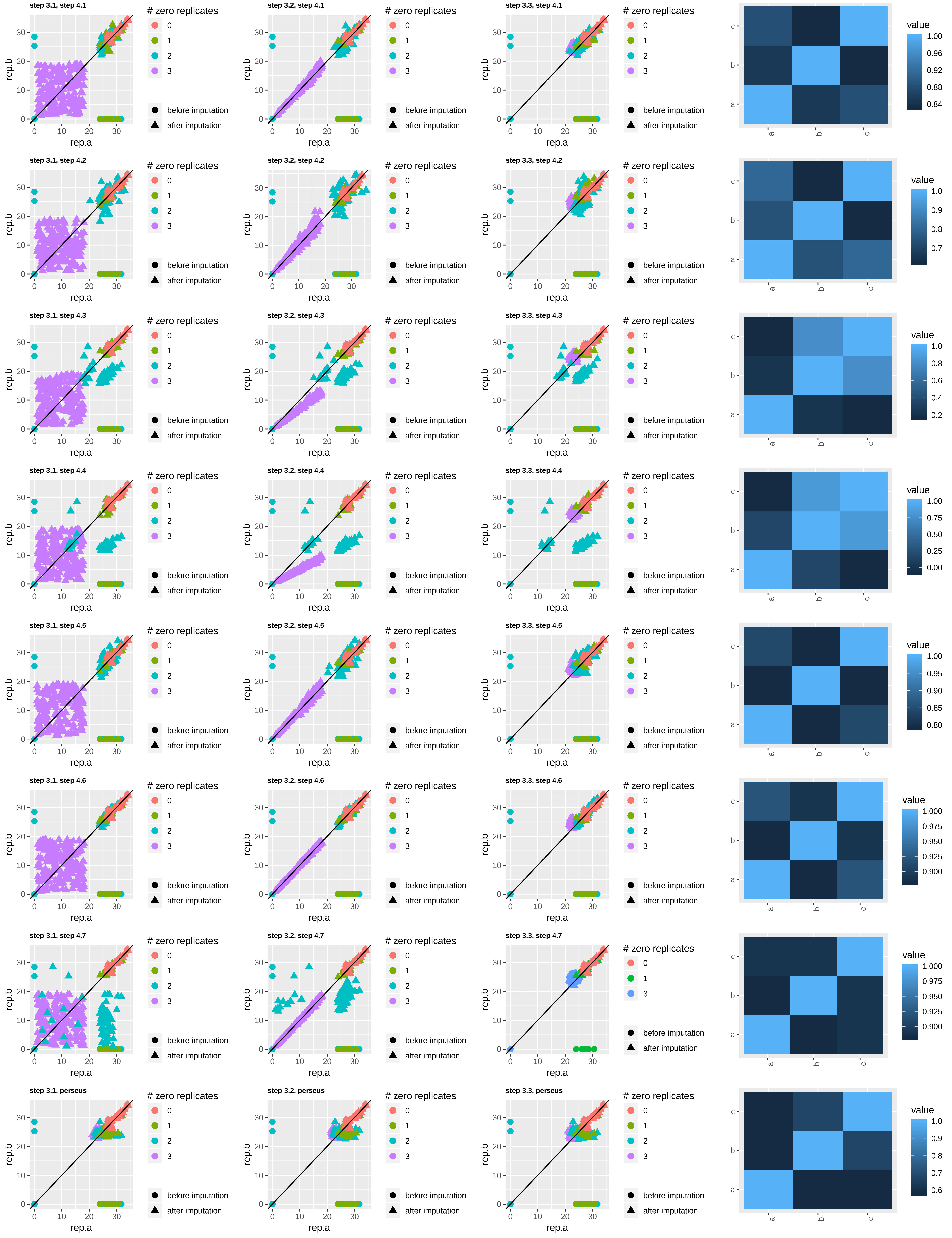

NCBP3−LAP, test tubes, condition 10
cases

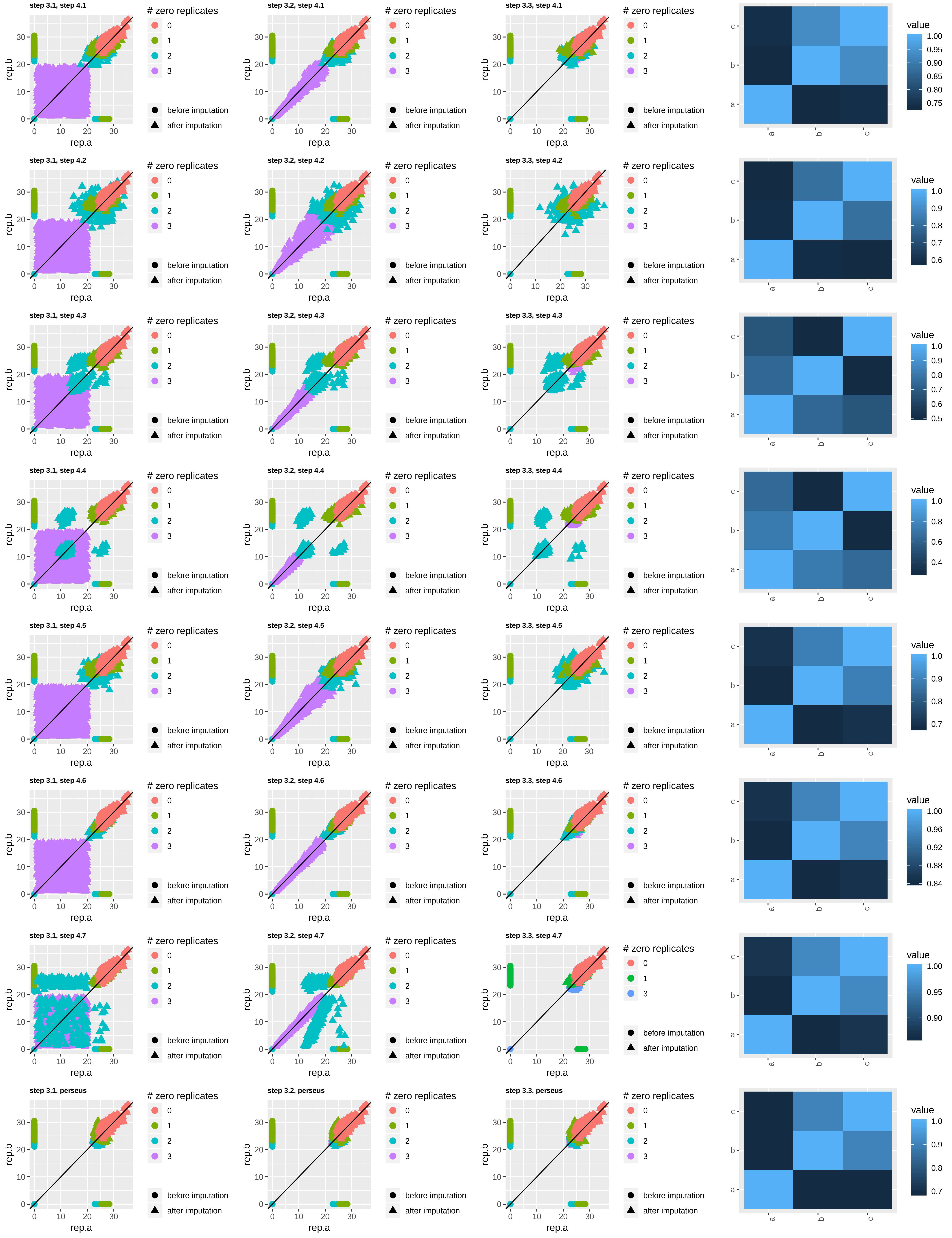

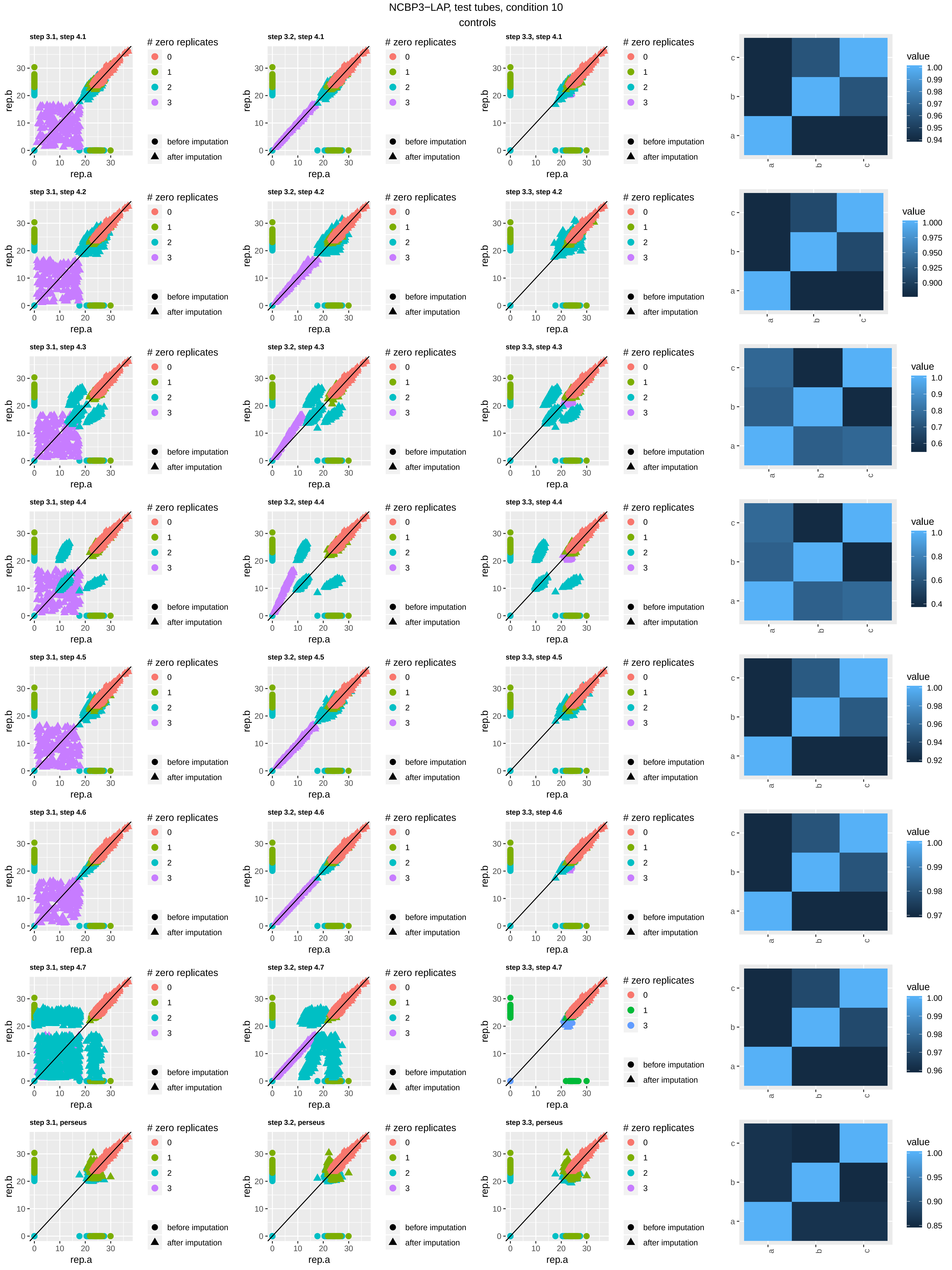

NCBP3−LAP, test tubes, condition 10
controls
step 3.1, step 4.1
step 3.2, step 4.1
step 3.3, step 4.1
step 3.1, step 4.2
step 3.2, step 4.2
step 3.3, step 4.2
step 3.1, step 4.3
step 3.2, step 4.3
step 3.3, step 4.3
step 3.1, step 4.4
step 3.2, step 4.4
step 3.3, step 4.4
step 3.1, step 4.5
step 3.2, step 4.5
step 3.3, step 4.5
step 3.1, step 4.6
step 3.2, step 4.6
step 3.3, step 4.6
step 3.1, step 4.7
step 3.2, step 4.7
step 3.3, step 4.7
step 3.1, perseus
step 3.2, perseus
step 3.3, perseus
rep.a
rep.b
zero replicates
0
1
2
3
before imputation
after imputation
value
a
b
c

NCBP3−LAP, test tubes, condition 12

cases

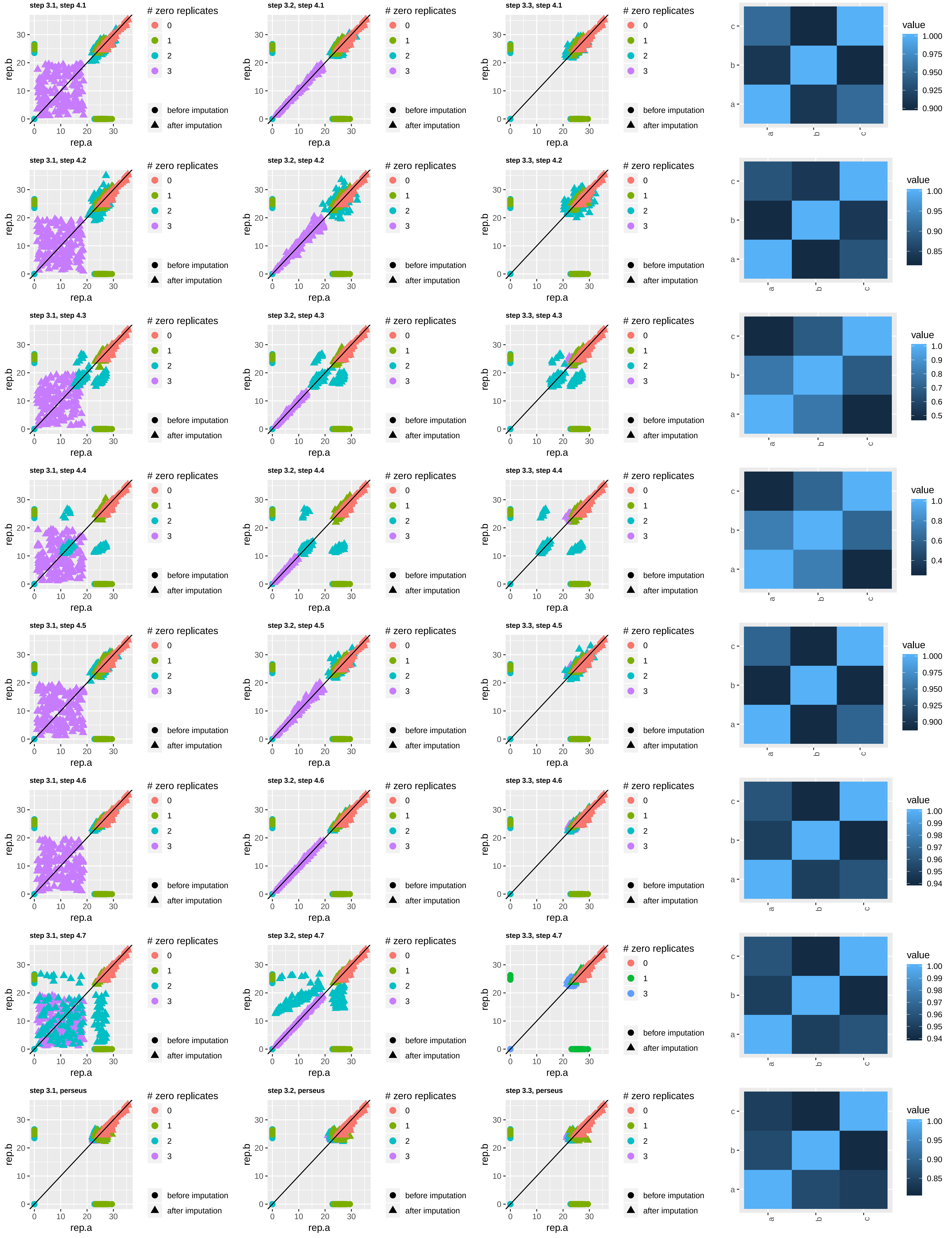

NCBP3−LAP, test tubes, condition 12

controls

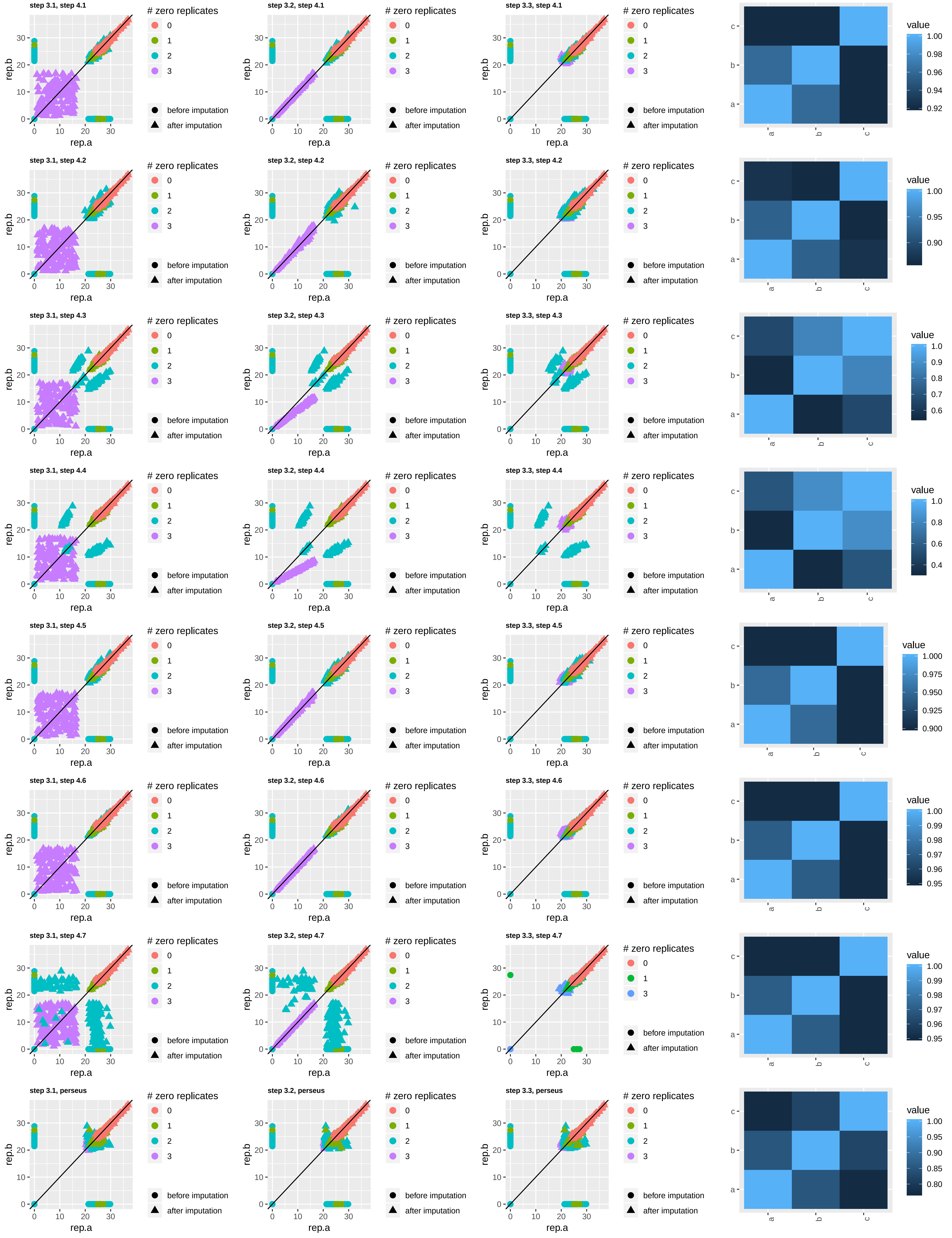

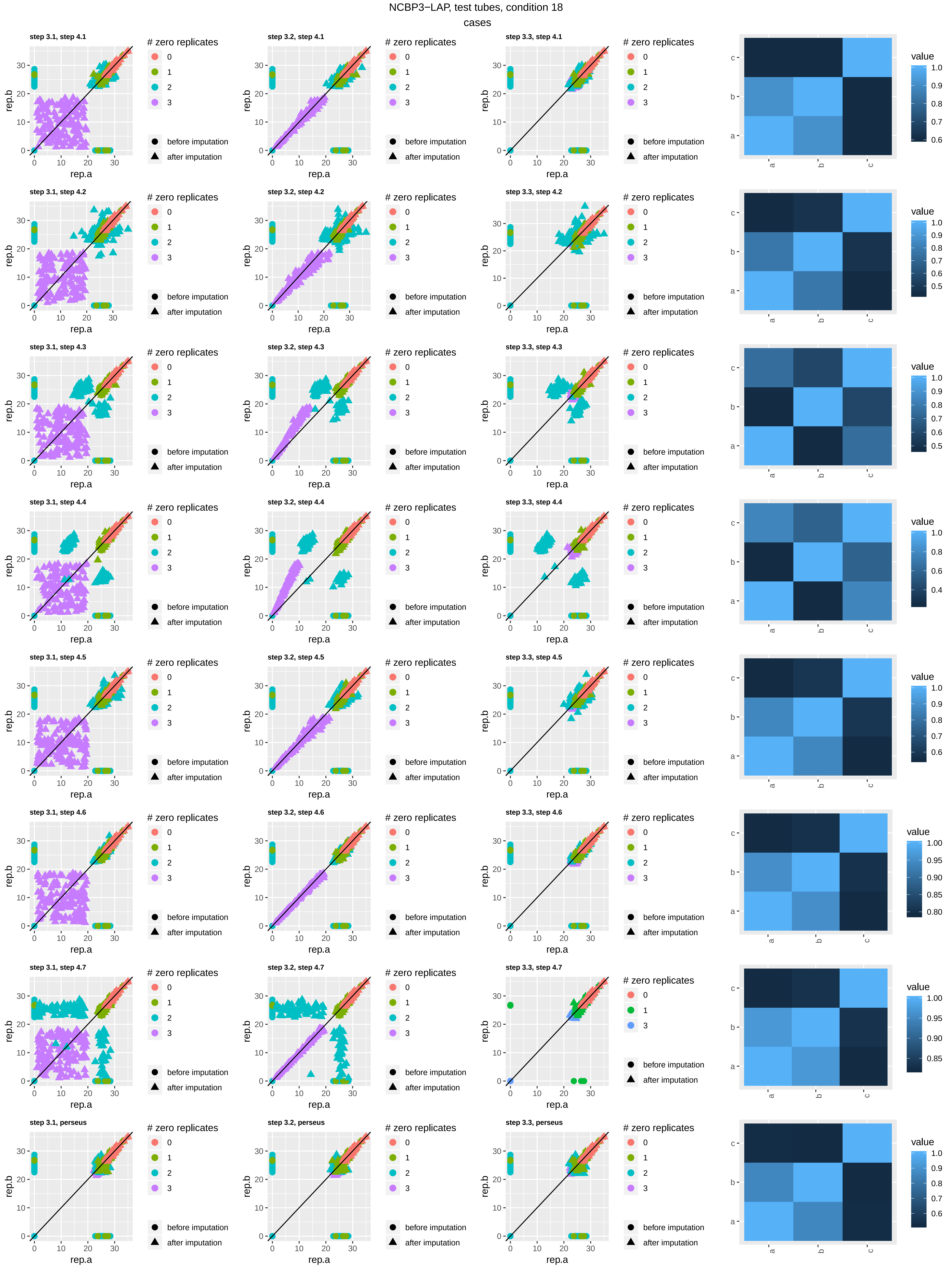

NCBP3−LAP, test tubes, condition 18
cases
step 3.1, step 4.1
step 3.2, step 4.1
step 3.3, step 4.1
step 3.1, step 4.2
step 3.2, step 4.2
step 3.3, step 4.2
step 3.1, step 4.3
step 3.2, step 4.3
step 3.3, step 4.3
step 3.1, step 4.4
step 3.2, step 4.4
step 3.3, step 4.4
step 3.1, step 4.5
step 3.2, step 4.5
step 3.3, step 4.5
step 3.1, step 4.6
step 3.2, step 4.6
step 3.3, step 4.6
step 3.1, step 4.7
step 3.2, step 4.7
step 3.3, step 4.7
step 3.1, perseus
step 3.2, perseus
step 3.3, perseus
rep.a
rep.b
zero replicates
0
1
2
3
before imputation
after imputation
value
a
b
c

NCBP3−LAP, test tubes, condition 18

controls

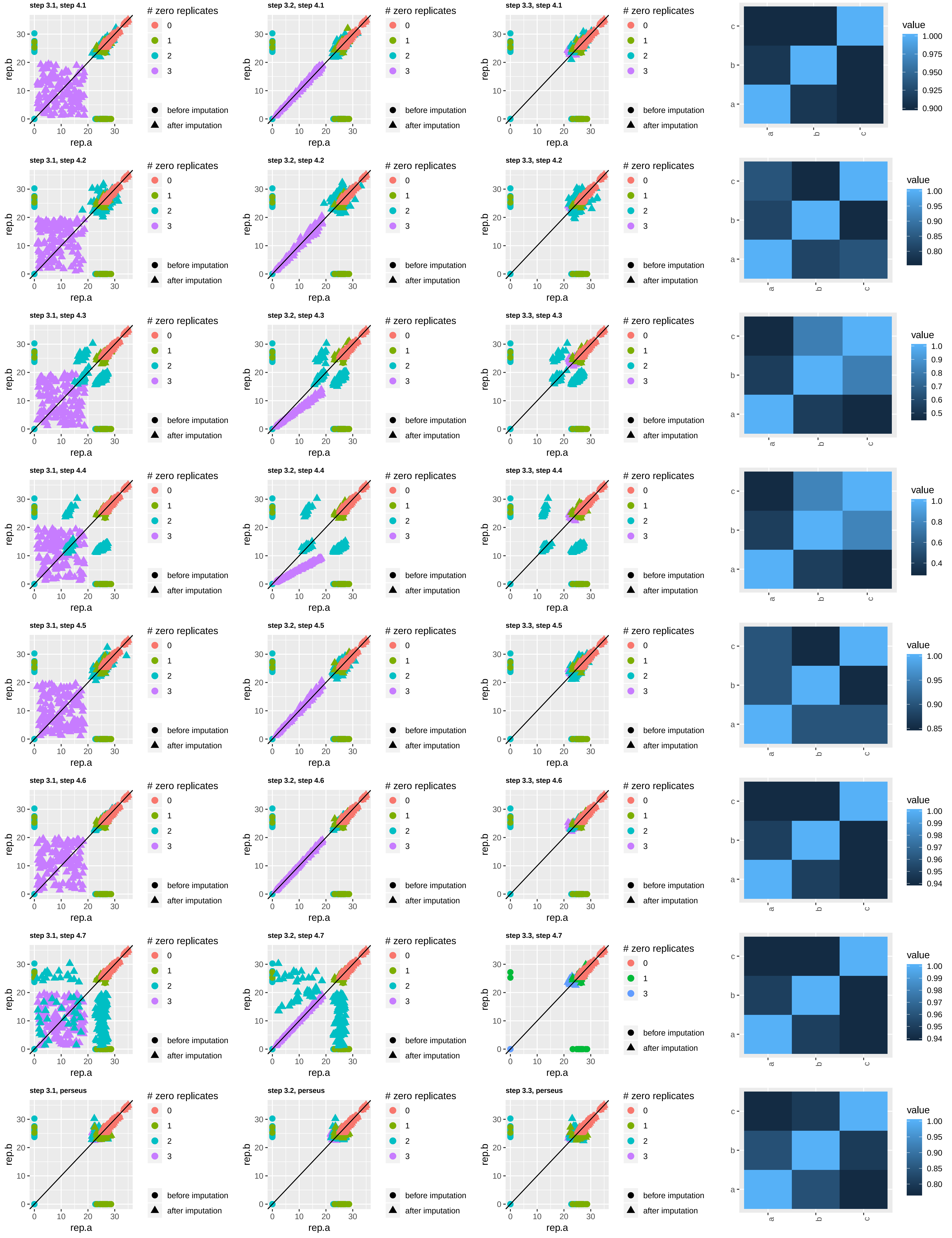

Supplement: gkaa743_Supplemental_Files [file gkaa743_supplemental_files.zip › Supplemental_materials.zip!/Supplemental_materials (1)/Supplemental_Data_replicate_correlation_plots.pdf]
