# Supplementary material for: Affinity proteomic dissection of the human nuclear cap-binding complex interactome
Source: Nucleic Acids Res. 2020 Sep 22;48(18):10456–69. doi: 10.1093/nar/gkaa743 (PMC7544204; doi:10.1093/nar/gkaa743)
Supplement: gkaa743_Supplemental_Files [file gkaa743_supplemental_files.zip › Supplemental_materials.zip!/Supplemental_materials (1)/RMSE_NRMSE_plots.zip!/ROC_AUC_combine/2019-06-14_20:03:44_roc_curves_iBAQ.pdf]

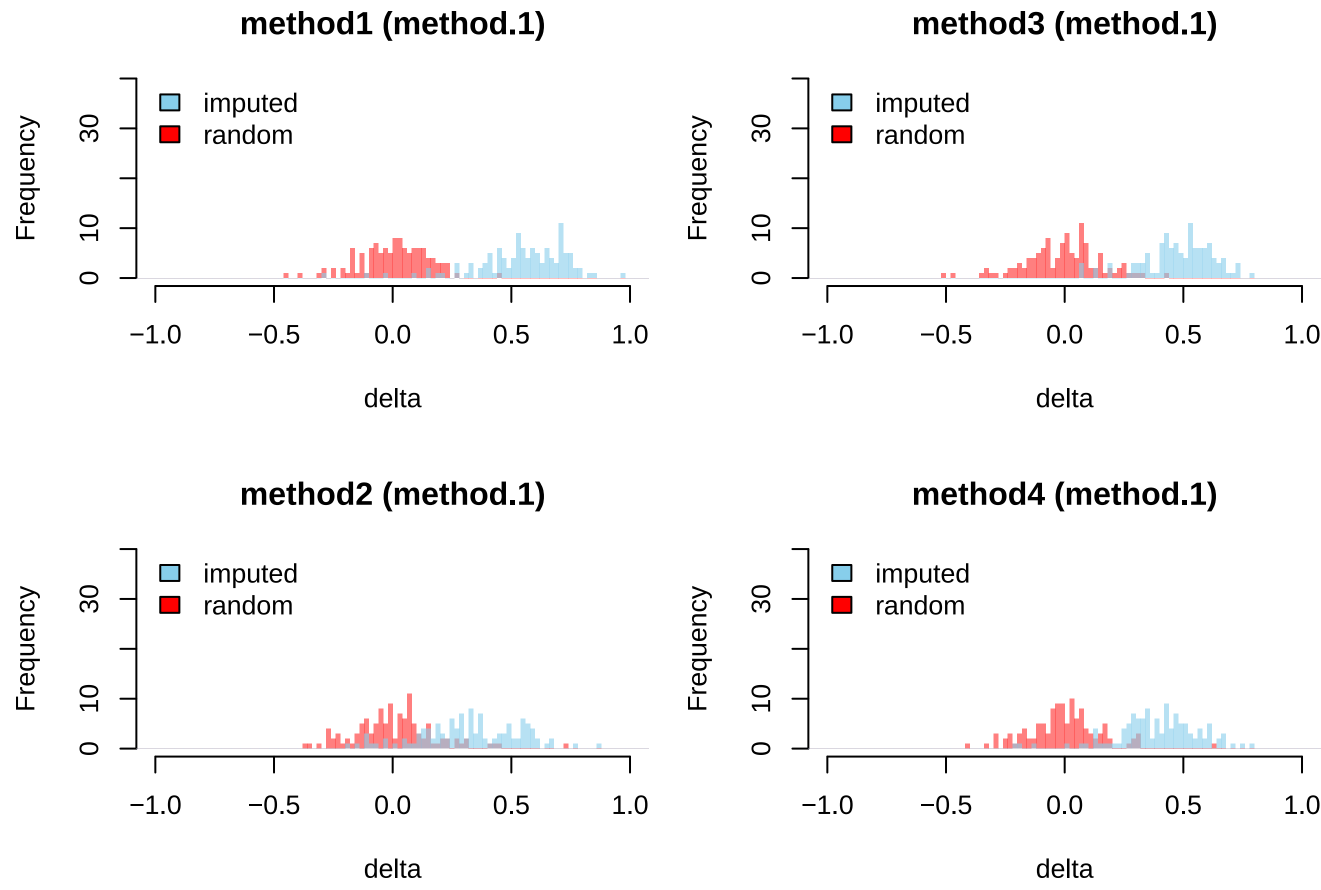
method1 (method.1)
method3 (method.1)
method2 (method.1)
method4 (method.1)
imputed
random
Frequency
0
10
30
−1.0
−0.5
0.0
0.5
1.0
delta

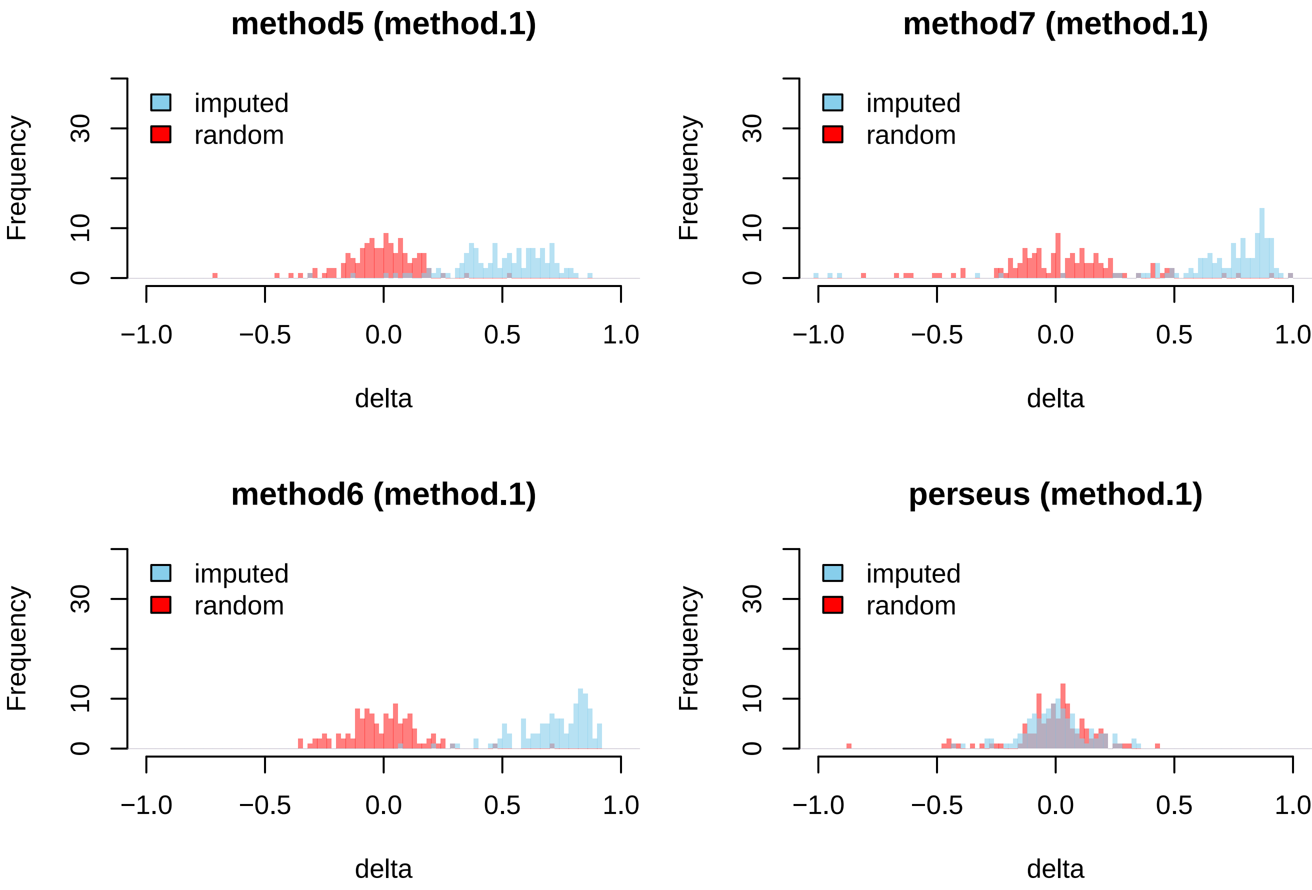
method5 (method.1)
method7 (method.1)
method6 (method.1)
perseus (method.1)
imputed
random
Frequency
0
10
30
−1.0
−0.5
0.0
0.5
1.0
delta

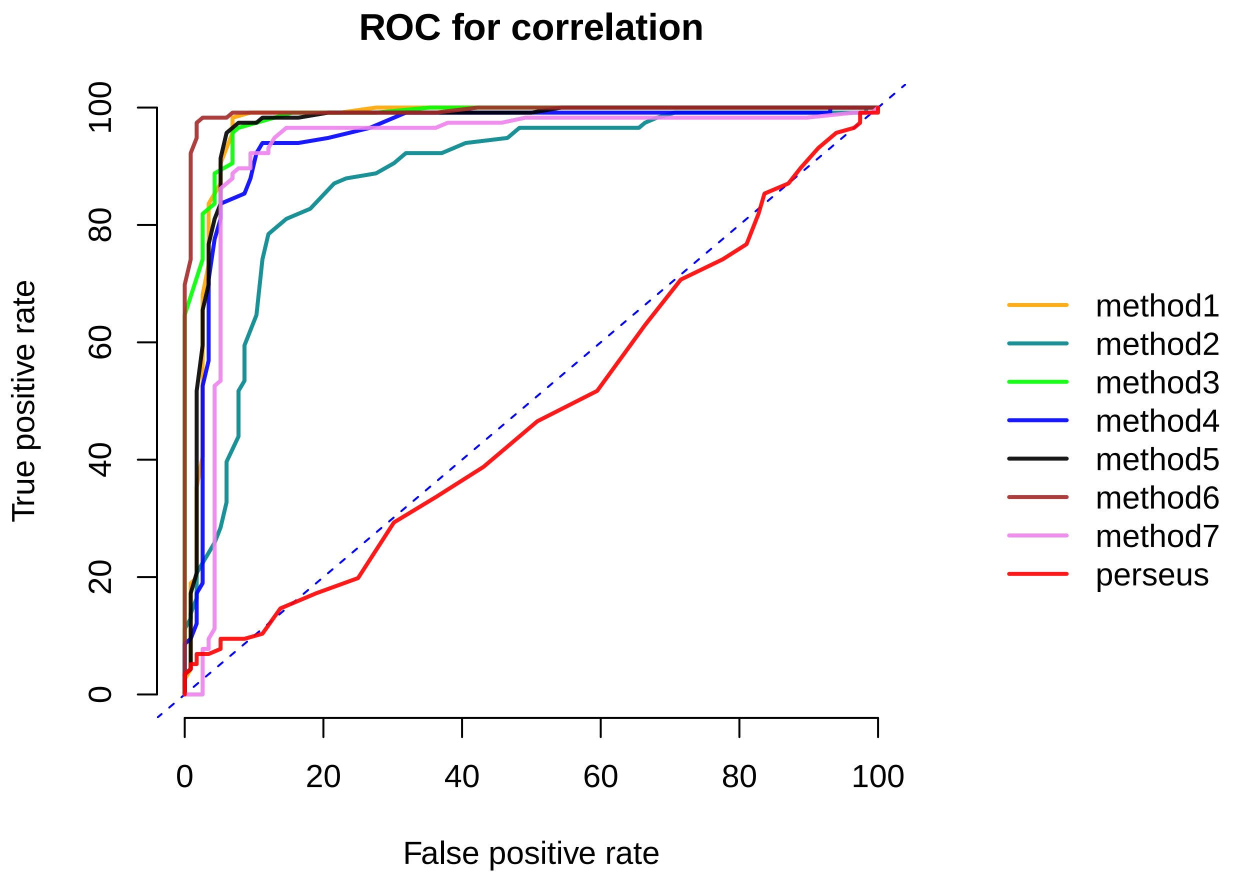

ROC for correlation
True positive rate
False positive rate
0
20
40
60
80
100
0
20
40
60
80
100
method1
method2
method3
method4
method5
method6
method7
perseus

	**auc**
method1	0.9714625
method2	0.8798306
method3	0.9830559
method4	0.9470125
method5	0.9686014
method6	0.9933487
method7	0.9273558
perseus	0.4804177
