# Supplementary material for: Affinity proteomic dissection of the human nuclear cap-binding complex interactome
Source: Nucleic Acids Res. 2020 Sep 22;48(18):10456–69. doi: 10.1093/nar/gkaa743 (PMC7544204; doi:10.1093/nar/gkaa743)
Supplement: gkaa743_Supplemental_Files [file gkaa743_supplemental_files.zip › Supplemental_materials.zip!/Supplemental_materials (1)/RMSE_NRMSE_plots.zip!/ROC_AUC_combine/2019-06-14_23:25:24_AUC_conf_intervals_iBAQ.pdf]

**method**	**AUC**
method6	0.99±0.05
method1	0.97±0.07
method3	0.97±0.07
method7	0.97±0.07
method5	0.96±0.08
method4	0.95±0.08
method2	0.89±0.11
perseus	0.5±0.13
